# Chemical Recycling of Poly(butylene adipate-*co*-terephthalate): A Glycolysis–Repolymerization Approach

**DOI:** 10.1021/acsomega.6c10047

**Published:** 2026-10-21

**Authors:** Cristian de Oliveira Romera, Alessandra da Rocha Duailibe Monteiro, Bruno Francisco Oechsler, Claudia Sayer, Caio Gomide Otoni, Pedro Henrique Hermes de Araújo

**Affiliations:** † Federal University of Santa Catarina, Campus Universitário Reitor João David Ferreira Lima, Florianópolis 88040-900, Santa Catarina, Brazil; ‡ Fluminense Federal University, Rua Passos da Pátria, n.156 bloco D sala 305, Niterói 24210-240, Rio de Janeiro, Brazil; § University of Campinas (Unicamp), Institute of Chemistry, Rua Monteiro Lobato, 270, Campinas 13083-862, São Paulo, Brazil; ∥ Graduate Program in Materials Science and Engineering (PPGCEM), Federal University of São Carlos (UFSCar), Rod. Washington Luís, km 235, São Carlos 13565-905, São Paulo, Brazil

## Abstract

Chemical recycling of bioplastics like poly­(butylene adipate-*co*-terephthalate) (PBAT) promotes a circular economy by overcoming mechanical recycling limitations. This study investigated PBAT glycolysis and subsequent repolymerization of crude glycolyzed products, integrated with an early-stage life cycle assessment (LCA) using OpenLCA software with the ReCiPe 2016 Midpoint (H) impact assessment method to anticipate potential environmental impacts and sensitivity of the system. Glycolysis was conducted at 150–190 °C using Sn­(Bu)_2_O (0.15 to 1.40 mol %) and NaHCO_3_ (5.0 mol %), presenting a Lewis acid coordination mechanism and a base-assisted glycolysis pathway, respectively, with ethylene glycol or 1,4-butanediol (4:1 molar ratio). Parameters including molar mass distribution (*M*_n_, *M*_w_, and *Đ*), species profile, and monomer yield were evaluated. Full conversion of the polymeric matrix was achieved within 2–5 h, leaving no detectable high-molecular-weight PBAT and yielding a mixture of oligomers and monomers, even at low catalyst concentrations (0.15 mol %), yielding oligomers with *M*_w_ of 0.6–1.4 kDa and up to 26.6 wt % monomer fraction. Notably, repolymerization without preliminary purification achieved *M*_w_ up to 10.6 kDa, comparable to 11.5 kDa reached using virgin raw materials. LCA results demonstrated improvements in 14 impact categories, with reductions between 6% and 35%. While fossil resource scarcity, ozone formation (human health and terrestrial ecosystem), and global warming potential increased due to the fossil-intensive energy baseline, the chemical proof of concept of directly utilizing crude glycolyzed products highlights the potential of this one-pot route to mitigate environmental burdens in polyester recycling, pending further property optimization.

## Graphical abstract

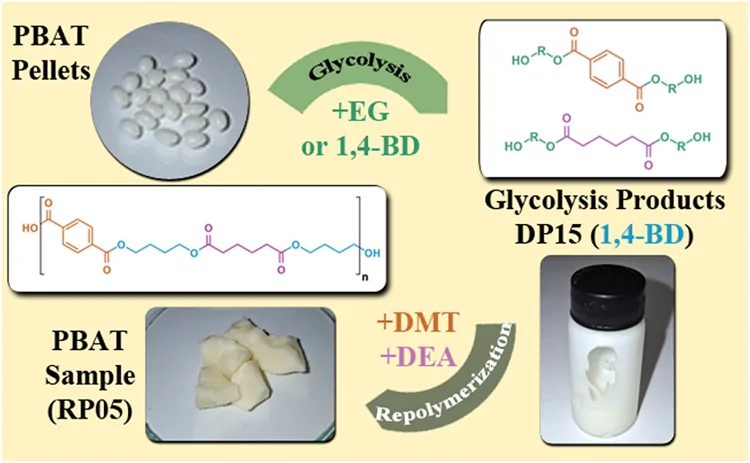

## Introduction

Recycling processes play a critical role in minimizing environmental impacts of materials. However, the global waste management landscape reveals that mechanical recycling alone is insufficient to address the growing volume of plastic waste. This limitation carries significant consequences, including increased government expenditure on waste disposal and accelerated pressure on landfill capacity.^1^ According to the OECD,^2^ the average recycling rate among member countries reached only 9% of the total plastic consumption, with European nations recording the highest rates at 14%. Although mechanical recycling is widely regarded as the most practical approach due to its technical feasibility and comparatively lower cost and environmental impact, it faces a fundamental challenge: the downgrading of material properties, which reduces both the commercial value and the quality of the recovered plastic.^3,4^

In recent years, chemical recycling has gained significant attention as a complementary strategy for mechanical recycling. It encompasses a series of processes in which macromolecules are broken down into smaller molecules, effectively converting plastics back to their fundamental building blocks, either monomers or oligomers.^5,6^ This approach is notably versatile: it enables the recovery of the original polymer through closed-loop recycling, the separation of components from polymer-based composites, and the synthesis of entirely new compounds via open-loop pathways.^5,6^

Poly­(butylene adipate-*co*-terephthalate) (PBAT) is a biodegradable polyester that has gained increasing relevance in the plastics industry as a material designed to minimize end-of-life environmental impact despite its fossil origin. Unlike conventional polyesters, such as poly­(ethylene terephthalate) (PET), PBAT undergoes biodegradation under both domestic and industrial composting conditions,^7^ making it a promising alternative to persistent plastic materials. It is synthesized via polycondensation of terephthalic acid, adipic acid, and 1,4-butanediol, typically in the presence of metal-based catalysts such as zinc-, tin-, or titanium-based compounds.^7^

The growing industrial relevance of bioplastics has prompted research into their chemical recycling, driven by the expectation that higher production volumes will accelerate their integration into existing recycling infrastructure.^4^ Several chemical recycling approaches have been evaluated for PBAT. For instance, the polymer was successfully depolymerized via cutinase enzymatic hydrolysis at 70 °C, followed by monomer purification and repolymerization achieved by converting terephthalic acid into dimethyl terephthalate and performing polycondensation with dibutyltin oxide as a catalyst.^8^ Other hydrolysis methods include the use of diphenyl phosphate (3.5 wt %) at 160 °C,^9^ as well as alkaline hydrolysis catalyzed by potassium hydroxide in combination with 1,4-butanediol (0 to 99 wt %) at temperatures up to 140 °C, at a polymer concentration of 0.04 g/mL, which produced dipotassium terephthalate and dipotassium adipate, targeted for the electro-oxidation of 1,4-butanediol.^10^ Regarding alcoholysis, PBAT has been chemically recycled via methanolysis (at 140 °C and 7 h) mediated by various metal-based catalysts (tin, zinc, or titanium), including dibutyltin­(IV) diacetate and dibutyltin oxide.^11^ The reaction mixture was distilled to remove methanol and recover the monomers, residual esters, and catalyst, which were subsequently repolymerized into PBAT without the addition of fresh catalyst.

Nevertheless, to the best of our knowledge, no study has specifically addressed the neutral glycolysis of PBAT, i.e., its depolymerization with diols (EG or 1,4-BD) mediated by mild, noncorrosive catalysts such as metal salts, in the absence of strong mineral acids or inorganic bases, despite this being one of the most widely employed strategies for the chemical recycling of PET. Unlike alkaline solvolysis methods (which form dipotassium/disodium salts requiring acidification steps), hydrolysis (which produces carboxylic acid and hydroxyl species), or methanolysis (which yields methyl-capped esters), neutral glycolysis directly generates predominantly hydroxyl-terminated oligomeric/monomeric esters. These −OH terminal groups retain nucleophilic activity, enabling continuous transesterification and rendering the crude product mixture directly suitable for seamless, one-pot step-growth repolymerization. Additionally, life cycle assessment (LCA) studies evaluating the environmental impacts of chemical recycling via chemolysis, which are fundamental for determining commercial and ecological viability, remain limited for PET applications,^12^ with even fewer investigations dedicated to bioplastics such as PLA.^13^

In the context of closed-loop chemical recycling, previous PBAT recycling strategies required intermediate processing or separation steps to isolate monomers prior to repolymerization.^8,11^ While alcoholysis approaches demonstrated repolymerization without fresh catalysts, they still relied on intermediate solvent removal.^11^ Therefore, the primary objective of this study was to investigate PBAT glycolysis in terms of depolymerization extent and to assess the chemical proof of concept of closed-loop repolymerization using the resulting mixture of monomers and oligomers without any intermediate purification steps, evaluated through structural recovery and molar mass restoration. The secondary objective was to conduct an early-stage screening LCA to anticipate potential environmental bottlenecks, evaluate the sensitivity of system boundaries and allocation choices, and benchmark this integrated route against conventional commercial PBAT production across key impact categories.

## Experimental Section

### Materials

PBAT (Table S1) was donated by Earth Renewable Technologies (ERT) in the form of oval-shaped pellets with average dimensions of 5.0 ± 0.2 mm (length), 3.4 ± 0.3 mm (width), and 2.5 ± 0.2 mm (height), measured across a sample of 30 pellets. Ethylene glycol (EG, 99%) and 1,4-butanediol (1,4-BD, 99%) were purchased from Sigma-Aldrich, along with the monomers diethyl adipate (DEA, 99%) and dimethyl terephthalate (DMT, 99%), and the catalysts titanium­(IV) butoxide (Ti­(OBu)_4_, 97%) and dibutyltin­(IV) oxide (Sn­(Bu)_2_O, 98%), while sodium bicarbonate (NaHCO_3_, 99%) was purchased from VETEC. All chemicals were used without further purification.

### Glycolysis Reactions

Glycolysis reactions were carried out in a 50 mL round-bottom flask using 5.00 g of PBAT pellets. Prior to experimentation, the commercially obtained PBAT pellets were stored sealed under vacuum in a desiccator at controlled room temperature (≤25 °C) to prevent moisture absorption and moisture-induced hydrolytic degradation. The glycol (1,4-BD or EG) was added at a 4:1 molar ratio to idealized PBAT repeating units (i.e., 1 BA + 1 BT) to maintain low melt viscosity and ensure efficient mixing. Since the repeating units of this random aliphatic-aromatic copolyester contain two transesterifiable ester groups each, or four together, and each glycol molecule provides two hydroxyl groups, half of the molecular weight of the idealized PBAT repeating unit (210 g·mol^–1^) was adopted as the effective molecular weight for stoichiometric calculations, noted as $\overset{®}{\mathrm{BAT}}$ . The catalysts employed were Sn­(Bu)_2_O (0.15 to 1.40 mol %) and NaHCO_3_ (5.0 mol %), with molar quantities calculated relative to $\overset{®}{\mathrm{BAT}}$ . Prior to heating, the reaction vessel was purged with a 2 min nitrogen purge, to displace atmospheric oxygen and establish an inert atmosphere in the headspace. The vessel was then hermetically sealed with a silicone stopper and operated as a closed batch reactor under autogenous pressure without continuous gas flow or volatile mass loss throughout the reaction. The detailed formulation parameters and the exact masses of all reactants for the glycolysis reactions are compiled in Supporting Information (SI) in Table S2. The flask, containing polymer, glycol, and catalyst, was placed in a sand bath under constant stirring and controlled heating in an IKA C-MAG HS 7 with an ETS-D5 controller. A warm-up period of 34 min was allowed for the system to reach the target temperature, after which isothermal conditions were maintained for 2 or 5 h, depending on the experimental condition (150, 170, or 190 °C). The glycolysis process is illustrated in Scheme 1. The run numbers indicate the chronological order in which the individual experiments (*n* = 1) were performed, following a randomized execution sequence to minimize systemic operational bias. Control runs (DP12 and DP13) were performed in duplicate (*n* = 2).

**Scheme 1:**
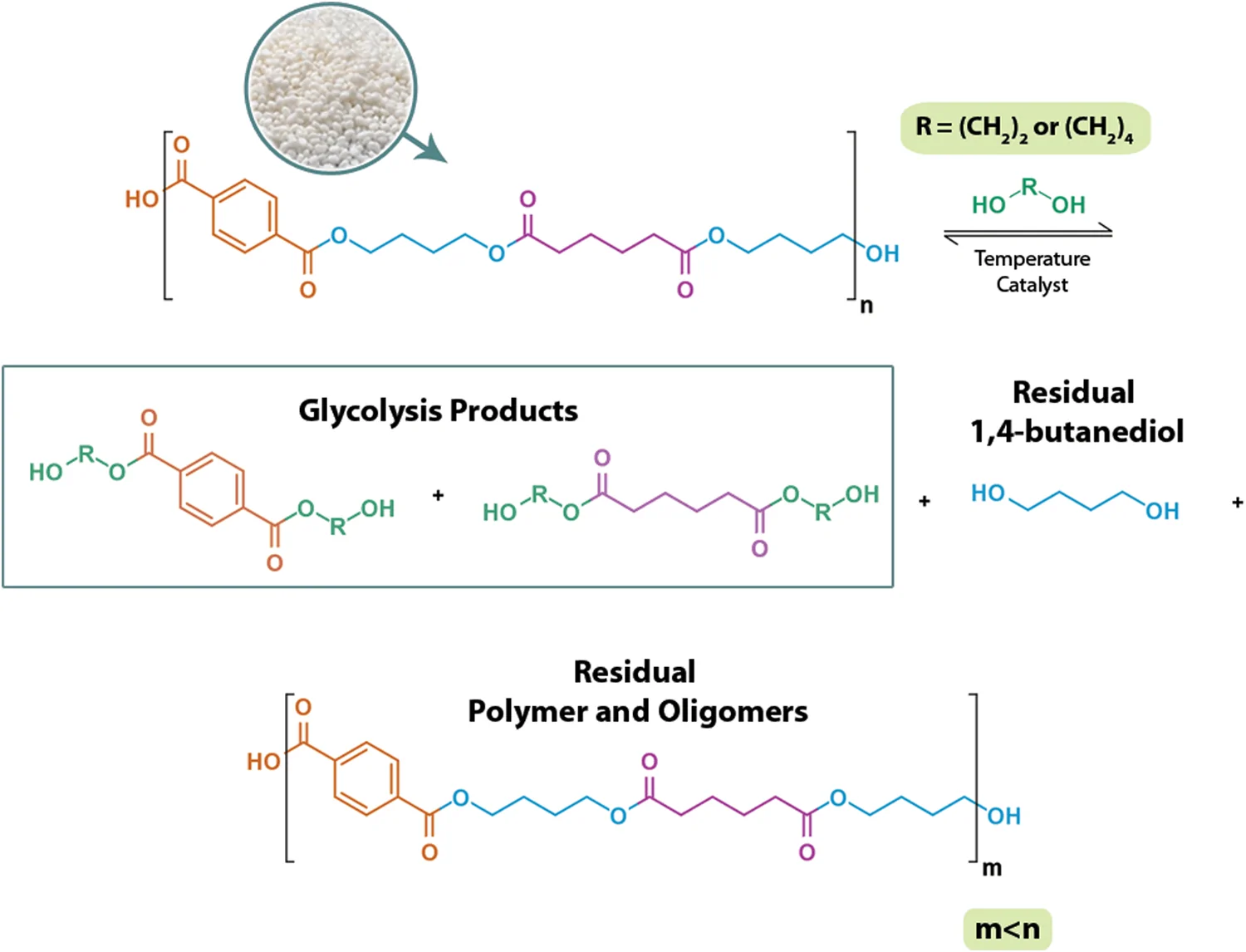
Glycolysis of PBAT Using Ethylene Glycol or 1,4-Butanediol as Glycolytic Agents in the Presence of Sn­(Bu)_2_O or NaHCO_3_ as Catalysts

### (Re)­Polymerization Reactions

Polymerization reactions were conducted in a closed system comprising a round-bottom flask coupled to a Dean–Stark trap and a condenser. Initially, 4.00 g of DMT was used, with the amount of DEA being varied to introduce a stoichiometric imbalance; the catalyst Ti­(OBu)_4_ was added at 0.2 mol % relative to the total monomer content, including glycol. For the repolymerization reactions, the catalyst Ti­(OBu)_4_ was added at 0.2 mol % relative to the total monomer content, and the equimolar amounts of DMT and DEA (1:1) were calculated relative to the 1,4-BD content, either based on the fractional amount present (fractional-batch feeding) or the total mass introduced (total-transfer feeding) during the preceding glycolysis steps. Stoichiometric calculations were based on a molar ratio of 2.1 mol of 1,4-BD per 2.0 mol of combined diesters (DMT and DEA) for both feeding strategies. For the total-transfer feeding, the reactants were added in the same flask used in the glycolysis reaction. The exact masses of all reactants for the polymerization and repolymerization reactions are compiled in SI by Tables S3 and S4, respectively. The flask containing monomers, glycol, and catalyst was placed in a sand bath, stirred, and subjected to controlled external heating in IKA C-MAG HS 7 with ETS-D5 controller. Prior to heating, the reaction vessel was purged with a 2 min nitrogen purge, to displace atmospheric oxygen and fill the headspace with an inert atmosphere. The system was then hermetically sealed with a silicone stopper, operating as a closed, batch reactor under autogenous pressure without continuous gas flow throughout the reaction. The reaction mixture was heated to 190 °C and maintained under a nitrogen atmosphere for 3.0–3.5 h, during which condensate was continuously collected in the Dean–Stark trap. Subsequently, a vacuum of 60 mmHg (80 mbar) was applied to facilitate further condensate removal, and the system was held at the target temperature (190 or 210 °C) for an additional 3.5 to 4.0 h. Finally, vacuum was maintained during cooling to prevent thermal oxidation and yellowing of the polymer. The residual excess glycol in the depolymerized stream was consumed as a reactive diol feedstock during the one-pot repolymerization step (by adjusting stoichiometry with virgin DMT/DEA streams), eliminating the need for intermediate glycol distillation.

### Characterization

#### Wide-Range Molar Mass Distribution, Polymer Fraction, and Subcalibration Fraction (SCF)

Wide-range gel permeation chromatography (W*R* GPC) analyses were performed using a Shimadzu LC-20A system equipped with a PLgel MiniMIX precolumn (5 μm, 50 × 4 mm) and two PLgel MiniMIX columns (5 μm, 250 × 4.6 mm) connected in series. Chloroform was used as the eluent at a flow rate of 0.3 mL·min^–1^ at 40 °C, with detection by a Shimadzu RID-10A refractive index detector. Molar mass distributions of glycolyzed products and repolymerized samples were characterized in terms of number-average molar mass (*M*_n_), weight-average molar mass (*M*_w_), and dispersity (*Đ*), determined from a calibration curve constructed with polystyrene standards in the range of 580 to 6,035,000 Da.

It should be noted that the theoretical molecular weights of the expected glycolysis monomers, namely, the double-substitution products of terephthalic and adipic acids with EG, are 254.24 and 234.25 Da, respectively, both falling below the lowest polystyrene standard used for calibration (580 Da). Consequently, the low-molar-mass fraction of the samples is not fully captured within the calibrated range. To account for this, the fraction eluting below 580 Da was a qualitative parameter defined as the subcalibration fraction (SCF), calculated as the percentage ratio of this subcalibration peak area to the total chromatographic area (i.e., the sum of the SCF area and the W*R* GPC integration area) for each sample, as illustrated in Figure S1 and presented in Table S5.

#### High-Resolution (HR) Low-Molar-Mass Distribution for Oligomeric Species

For glycolyzed samples exhibiting higher degrees of conversion, a precise characterization of HR low molar mass species by GPC (HR GPC) was performed using a Waters Styragel HR 0.5 column (5 μm, 300 × 7.8 mm), under the same instrumental conditions described above. Calibration was performed using some compounds and polystyrene standards in the range of 90 to 2000 Da (Figure S2). Additionally, a reference standard of one of the expected glycolysis products, bis­(2-hydroxyethyl) terephthalate (BHET, 254.24 Da), was included as a calibration point. The area correction based on the refractive index increment (d*n*/d*c*) of each species was performed using the Lorentz–Lorenz equation (eqs S1–S3). The resulting corrected refractive factors (*f*) for each oligomeric species, along with their composition-based averages, are presented in Table S6 and eqs S4–S6, respectively. The average corrected refractive factors were multiplied by the HR GPC integration area of each oligomeric fraction to obtain the mass fraction of species. The corrected areas are presented in Table S7, whereas the estimated mass fractions of monomers and oligomers are reported in Table S8.

#### BHET Yield

High-Performance Liquid Chromatography (HPLC) was performed using a Shimadzu LC-2050C system equipped with a Supelcosil LC-18 Supelguard precolumn (5 μm, 20 × 4 mm) and a Supelcosil LC-18 analytical column (5 μm, 250 × 4.6 mm). The mobile phase consisted of water (25%) and methanol (75%) at a flow rate of 0.8 mL·min^–1^ (25 °C), with UV detection at 254 nm. BHET was quantified against an external calibration curve constructed from standard solutions at 50 to 1500 mg·L^–1^ (*R* ^2^ = 0.9997). The BHET yield was calculated as the ratio of the experimentally determined concentration to the theoretical concentration expected from stoichiometry, expressed as a percentage. The stoichiometric calculations were presented in SI by eqs S7–S9.

#### Profile of Obtained Esters, Residual of DMT/DEA, and Composition of Comonomers

Proton Nuclear Magnetic Resonance (^1^HNMR) spectra were recorded on a Bruker AVANCE DPX 400 MHz spectrometer using CDCl_3_ as solvent. Chemical shifts (δ) are reported in parts per million (ppm) relative to tetramethylsilane (TMS, δ = 0.00 ppm) as the internal reference. The conversion of internal ester chains (*X*_IEC_) was calculated using eq 1, where *I*_T_ was the terephthalate peak integration at 8.1 ppm, while *I*_TIC_ and *I*_AIC_ were the peak integrations of terephthalate and adipate internal chains at 4.43 and 4.15 ppm, respectively.

1 $$X_{IEC}=\left[1-\frac{{\left(\frac{I_{\mathrm{TIC}}+I_{\mathrm{AIC}}}{I_{T}}\right)}_{sample}}{{\left(\frac{I_{\mathrm{TIC}}+I_{\mathrm{AIC}}}{I_{T}}\right)}_{PBAT}}\right]\times 100$$

For glycolysis products, peak integration was also used to assess the qualitative ester distribution. Each individual peak integration (IP) was normalized to the sum of adipate and terephthalate contributions (IAT), taken from the characteristic signals at 2.4 and 8.1 ppm, respectively. The normalization is represented through eqs S10 and S11 in the SI. This normalization minimizes the influence of sample heterogeneity arising from differences in glycol composition. Residual monomer (DMT and DEA) content in the repolymerization mixtures was quantified by comparing specific proton resonances as presented in the SI by eqs S12–S15. The composition of PBAT samples in terms of monomers was determined by molar relation between terephthalate (8.1 ppm) and adipate (2.4 ppm) contributions, discounting free DMT and DEA.

#### Thermal Characterization

Differential Scanning Calorimetry (DSC) measurements were performed on a PerkinElmer DSC 6000 under a N_2_ atm. Samples (∼2–3 mg) were subjected to two heating cycles from −40 to 200 °C at a rate of 20 °C min^–1^, with an intermediate controlled cooling step between runs. The crystallization temperature (*T*_c_) and melting temperature (*T*_m_) were determined from the peak maxima of the crystallization exotherm and melting endotherm, respectively. The percent crystallinity (*X*_c_) of the polymer samples was calculated using eq 2, where Δ*H*_m_ is the specific melting enthalpy of the sample, Δ*H*_m_° is the melting enthalpy of the 100% crystalline PBAT (114 J/g),^14^ and w represents the mass fraction of polymer sample based on ^1^H NMR.

2 $$X_{c}=\left(\frac{\Delta H_{m}}{\Delta H_{m}^{{}^{\circ}}\times w}\right)\times 100\%$$

#### LCA

The LCA aimed to quantify the environmental impacts associated with PBAT production and to assess how the use of monomers/oligomers derived from glycolysis affects these impacts, identifying mitigation potential and opportunities for improvement in an early-stage screening concept. Calculations were based on a functional unit of 1 kg of PBAT with system boundaries defined using a cradle-to-gate approach. Since the study focused on PBAT pellets, downstream processing, consumer use, and end-of-life stages were excluded from the model. Catalysts are conventionally excluded from the inventory input due to the cutoff rule (<1 wt % threshold). Adipic and terephthalic acids were chosen as the primary raw materials, rather than the esters used experimentally, due to these specific diesters being unavailable as fully inventory-validated data sets in the Ecoinvent database. Three distinct scenarios were evaluated to compare PBAT production from virgin raw materials against repolymerization of glycolysis products (Figure S2): conventional PBAT production from virgin raw materials (case A); PBAT production incorporating monomers/oligomers from 1,4-BD glycolysis, including the primary production environmental impacts (case B, conservative allocation model); and PBAT production from glycolysis products, applying a zero-burden cutoff assumption for the input waste resin (case C, standard recycling model).

LCA modeling was performed in OpenLCA 2.2.0 software with the ReCiPe 2016 Midpoint (H) impact assessment method and the Ecoinvent database (ver. 3.6). Environmental impact data for fossil-based PBAT production were obtained from Luo et al.,^15^ who compiled data from industrial manufacturers and environmental impact assessment reports (Table S9). Given the limited availability of LCA studies specifically addressing glycolysis, the process was modeled by adapting input data from Iturrondobeitia et al.,^12^ in which key parameters from PET glycolysis with propylene glycol, including consumption of electricity, natural gas, and water, were adjusted to reflect glycolysis conducted with 1,4-BD (Table S10). The transportation distance for all feedstocks to the processing facility was set at a baseline of 500 km. This represents a conservative regional logistics scenario, ensuring that the model remains applicable to larger, highly industrialized nations, where supply chains are geographically distributed.^15,16^ The (re)­polymerization thermal regime reflects standard commercial polycondensation parameters. All direct material inputs for Cases B and C were derived from experimental mass yields (Tables S9 and S10), whereas process energy requirements were modeled based on industrial-scale thermal duty for equivalent polycondensation stages.

## Results and Discussion

### Glycolysis Reactions

To establish a baseline for evaluating the glycolysis reactions, commercial PBAT was characterized by DSC, ^1^HNMR, and GPC, with the corresponding thermogram and spectra presented in Figure S4a–c. These baseline parameters are subsequently revisited throughout the text to benchmark reaction progress and product properties. The ^1^HNMR spectrum (Figure S4b) was consistent with pure PBAT, showing no peak around 5 ppm, which confirms the absence of blending with polyesters, such as poly­(lactic acid), in the commercial sample. Furthermore, DSC analysis (Figure S4a) revealed a single melting temperature near 120 °C, typical of pure, commercial-grade PBAT. The PBAT was applied in glycolysis reactions as-received to mimic real-world recycling conditions.

The characterization of glycolysis products is presented in Table 1.

**Table 1:** Chemical and Structural Characterization of Glycolysis Products Obtained at a 4:1 Glycol: $\overset{®}{\mathrm{BAT}}$ Molar Ratio, Including GPC Data (Both Methods) and BHET Yield

Sample^a^	*T* (°C)	*t* (h)	glycol	Cat.	Cat. (mol %)	WR *M*_n_ (kDa)^b^	WR *M*_w_ (kDa)^b^	PBAT fraction (wt %)^b^	*X*_IEC_ (%)^c^	HR *M*_n_ (kDa)^d^	HR *M*_w_ (kDa)^d^	BHET yield (%)^e^
PBAT	-	-	-	-	-	41.74	91.85	-		-	-	-
DP03	150	5	EG	Sn(Bu)_2_O	0.70	0.50	0.59	0.0	72.3	0.44	0.62	14.8
DP14	150	5	EG	Sn(Bu)_2_O	0.35	0.51	0.58	0.0	45.0	0.47	0.69	^f^
DP15	150	1	1,4-BD	Sn(Bu)_2_O	0.35	1.38	2.62	15.7	36.8	-	-	-
DP11	150	1	EG	Sn(Bu)_2_O	0.35	1.23	11.27	24.4	26.7	-	-	-
DP10	150	5	EG	Sn(Bu)_2_O	0.15	0.68	0.93	0.0	40.7	0.79	1.44	^f^
DP05	170	2	EG	Sn(Bu)_2_O	1.40	0.46	0.51	0.0	70.5	0.44	0.73	13.8
DP06	170	2	EG	Sn(Bu)_2_O	0.70	0.44	0.48	0.0	72.5	0.43	0.67	14.9
DP04	170	5	1,4-BD	Sn(Bu)_2_O	0.70	0.52	0.61	0.0	73.1	0.59	0.80	-
DP01	170	5	EG	Sn(Bu)_2_O	0.70	0.52	0.63	0.0	73.1	0.43	0.63	15.5
DP12	170	5	EG	Sn(Bu)_2_O	0.43	0.56	0.69	0.0	74.5	0.42	0.60	14.2
DP13	170	5	EG	Sn(Bu)_2_O	0.43	0.50	0.59	0.0	69.4	0.42	0.59	14.1
DP08	170	5	1,4-BD	NaHCO_3_	5.00	0.48	0.54	0.0	66.7	0.60	0.85	-
DP07	170	5	EG	NaHCO_3_	5.00	0.46	0.52	0.0	69.4	0.44	0.68	12.7
DP02	190	5	EG	Sn(Bu)_2_O	0.70	0.46	0.62	0.0	71.4	0.43	0.61	18.0
DP09	190	5	EG	Sn(Bu)_2_O	0.15	0.53	0.80	0.0	71.5	0.42	0.62	14.3

a All reactions correspond to single independent runs (*n* = 1) except for control runs DP12 and DP13, which were performed in duplicate (*n* = 2).

b Based on a GPC calibration curve with a lower detection limit of 580 Da (WR GPC).

c Determined by ^1^HNMR.

d GPC run with a column suitable for low-molar-mass compounds and calibrated with low-molar-mass standards (HR GPC).

e Determined by HPLC.

f Low yield into monomers was estimated from GPC peak areas obtained by method (b).

For the quantification and molar mass determination of the oligomeric fraction via HR GPC, the response constant of the differential refractive index detector depends directly on the specific refractive index increment (d*n*/d*c*). Unlike high-molar-mass polymers, where the d*n*/d*c* value is virtually constant, the d*n*/d*c* of oligomers varies with the degree of polymerization due to the volumetric and electronic contributions of the end groups. This variation can be theoretically modeled and corrected based on molar refractivity as described by the Lorentz–Lorenz equation,^17,18^ adjusting the detector signal to prevent errors in peak area integration and molar mass distribution profiling.^19^ This methodology allows HR GPC peak areas to become directly proportional to mass; thus, the corrected relative peak area (wt %) represents the mass fraction directly, enabling meaningful comparison across the different HR GPC runs (Table S8). The data are organized in the order of increasing temperature and decreasing catalyst concentration.

All reactions, but those carried out for only an hour (DP11 and DP15), allowed PBAT depolymerization. GPC confirmed the absence of high-molar-mass peaks after 2 and 5 h, indicating extensive polymer degradation and the complete consumption of the high-molar-mass PBAT fraction into lower-molar-mass species through glycolysis with EG and 1,4-BD. Figure 1 highlights the influence on glycolysis outcomes of key process variables, such as temperature, catalyst concentration, and reaction time.

**Figure 1:**
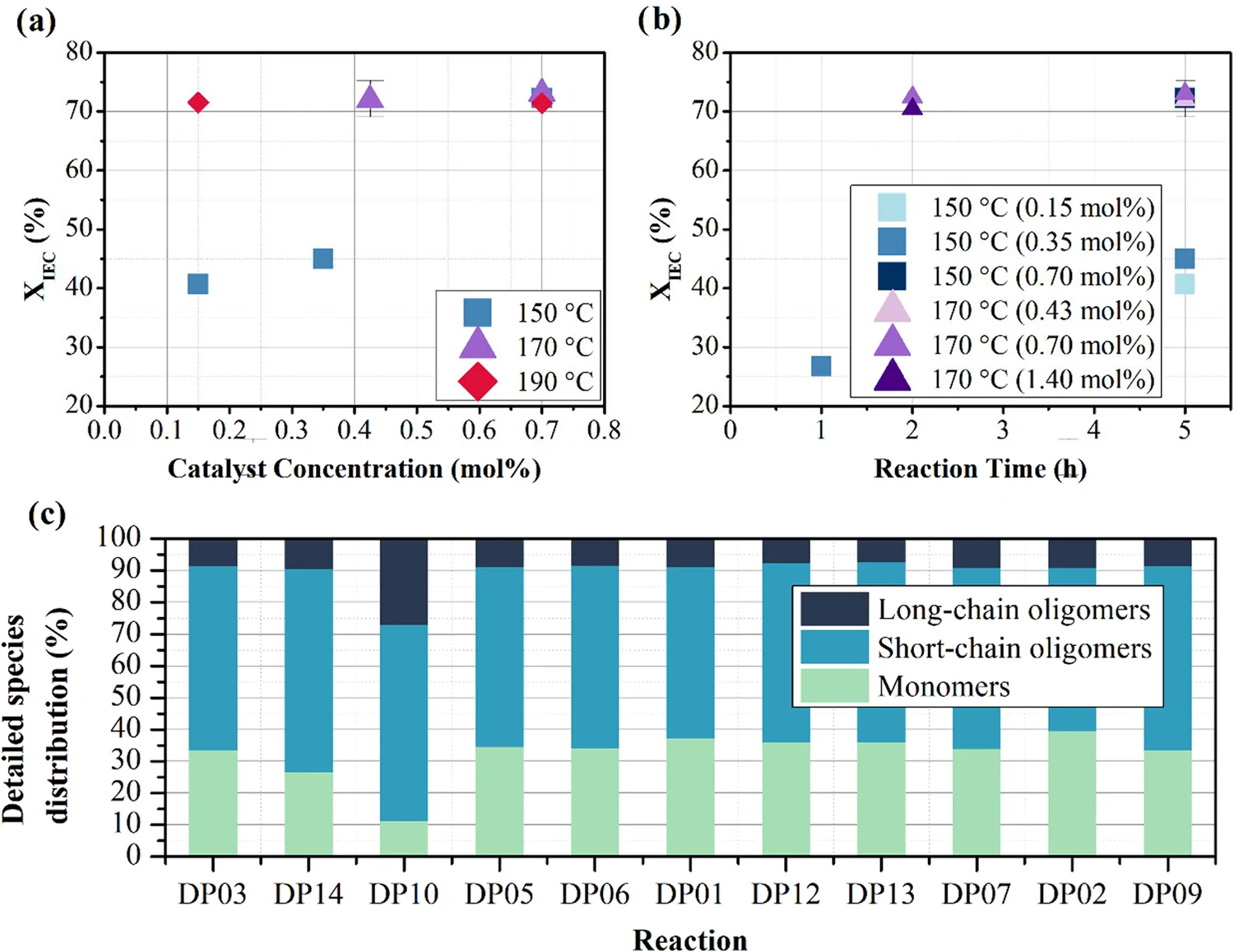
Conversion of internal ester chains (determined as *X*_IEC_ by ^1^HNMR) for EG-based glycolysis reactions as a function of (a) Sn­(Bu)_2_O catalyst concentration (at 5 h reaction), (b) reaction time, and (c) detailed species distribution determined by HR GPC and its Lorentz–Lorenz correction.

Additionally, Figure 2 shows the reduction in peak intensity within the 4.0–4.6 ppm region, which corresponds to the consumption of internal ester linkages from both terephthalate and adipate units. This behavior was used to calculate the conversion of internal ester chains (*X*_IEC_).

**Figure 2:**
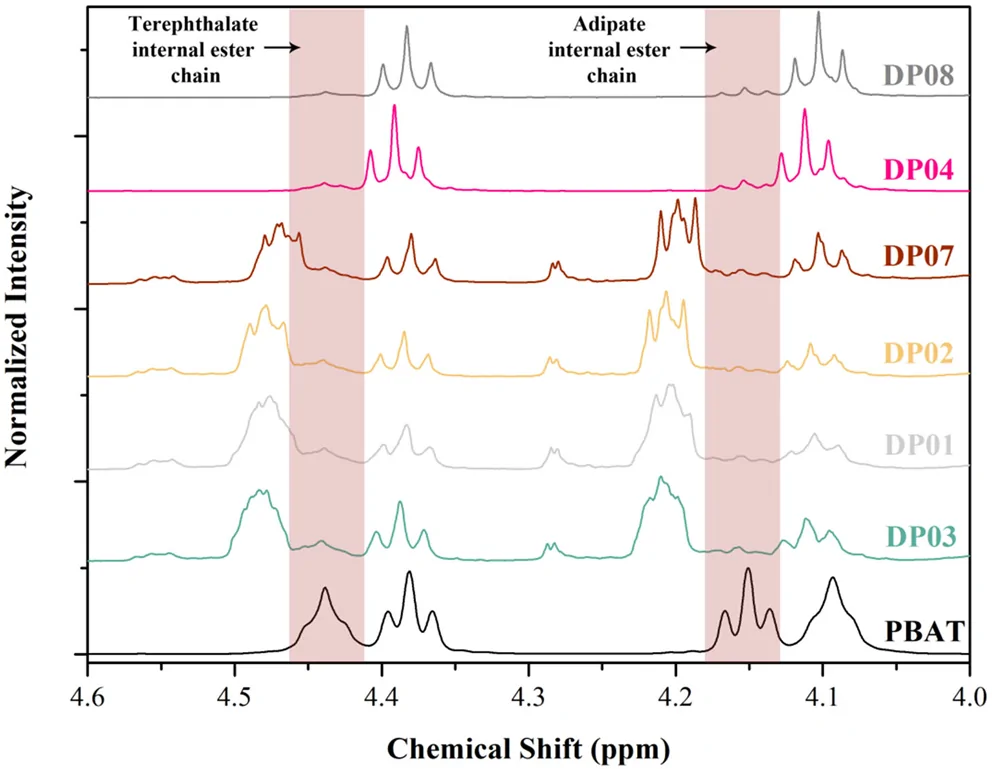
^1^HNMR of some glycolysis products in the region 4.0 to 4.6 ppm, showing the consumption of internal ester linkages.

Figure 1a shows the relationship between *X*_IEC_ and catalyst concentration at different temperatures. In the Lewis acid activation mechanism for organotin catalysts, the Sn atom coordinates with the carbonyl oxygen of the ester group, increasing its electrophilicity and rendering it highly susceptible to glycol cleavage during glycolysis, as well as promoting the reverse transesterification (polycondensation) during the repolymerization phase under driving conditions.^11,20^ At 150 °C, catalyst concentrations of 0.70, 0.35, and 0.15 mol % yielded HR *M*_w_ values of 0.62 (DP03), 0.69 (DP14), and 1.44 kDa (DP10), with *X*_IEC_ of 72.3, 45.0, and 40.7%, respectively (Table 1). The influence of catalyst concentration is further reflected in the monomer content, which decreased from 33.4 to 26.6 and 11.4 wt % as catalyst loading was reduced (Figure 1c). At 190 °C, reducing the catalyst concentration from 0.70 to 0.15 mol % showed similar HR *M*_w_ of 0.62 (DP02) to 0.61 kDa (DP09), accompanied by similar *X*_IEC_ values of 71.5 and 71.4%, respectively. In terms of monomer content, the differences between these conditions were minimal, with BHET yields of 18.0 and 14.3 wt %, respectively, suggesting that at 190 °C, the influence of catalyst concentration is not much pronounced on the formation of low molar mass species. Despite having virtually identical HR *M*_w_ values, the detailed distribution revealed a slightly higher contribution of long-chain oligomers in DP02. This can be attributed to competition between glycolysis and repolymerization, a consequence of the dual transesterification activity of Sn­(Bu)_2_O,^11^ even though overall molar masses remained low due to the stoichiometric imbalance imposed by the excess of glycol.

Figure 1b illustrates the synergistic effect of reaction time on glycolysis conversion under milder conditions (150 and 170 °C) and different catalyst concentrations. At 150 °C with 0.35 mol % catalyst, the *X*_IEC_ increased markedly from 26.7% at 1 h (DP11) to 45.0% at 5 h (DP14), with DP11 still retaining a residual PBAT fraction of 24.4 wt %, indicating incomplete conversion at shorter reaction times despite the stoichiometric excess of glycol. A similarly low conversion after 1 h was observed for the equivalent reaction conducted with 1,4-BD (DP15), which exhibited a PBAT fraction of 15.7 and a *X*_IEC_ of 36.8%.

At a higher catalyst concentration (0.7 mol %) and 170 °C, shortening the reaction time from 5 to 2 h led to similar *X*_IEC_ values of 73.1% (DP01) and 72.5% (DP06). This subtle difference can be attributed to the higher fraction of short-chain oligomers in DP06, while fractions of long-chain oligomers, monomer, and BHET yield were higher in DP01, consistent with the longer reaction time allowing further depolymerization. These results again reflect the dual activity of the transesterification catalyst, which simultaneously promotes depolymerization and repolymerization, favoring monomer formation while retaining a fraction of long-chain oligomers.

As shown in Figure 1b, a comparison of 0.70 (DP06) and 1.40 mol % (DP05) catalyst concentrations at 170 °C over 2 h demonstrates that doubling the catalyst loading had a negligible effect on the *X*_IEC_ (72.5 and 70.5%, respectively). Consistent with this, monomers and BHET yields remained nearly identical across both conditions.

The influence of temperature was assessed at a catalyst concentration of 0.70 mol % over 5 h of reaction (Table 1 and Figure 1a,c). Increasing the temperature from 150 to 170 and 190 °C resulted in similar *X*_IEC_ (72.3, 73.1, and 71.4%, respectively), and a slight increase in monomers (33.4, 37.3, and 39.6 wt %, respectively), while short-chain oligomers decreased (58.0, 53.8, and 51.4 wt %). The BHET yields were 14.8, 15.5, and 18 wt %, respectively, corroborating that higher temperatures favor monomer recovery during glycolysis.

The influence of the diol type on the catalytic system for glycolysis was examined by comparing EG- and 1,4-BD-mediated reactions under two different catalytic systems: 0.70 mol % Sn­(Bu)_2_O (DP01 and DP04) and 5.00 mol % NaHCO_3_ (DP07 and DP08). NaHCO_3_ was previously applied in PET waste glycolysis as an effective and environmentally friendly catalyst for the production of BHET.^21^ In contrast to Sn­(Bu)_2_O, NaHCO_3_ acts as a mild basic cocatalyst by promoting the deprotonation of glycol hydroxyl groups. This generates a more nucleophilic alkoxide species (in situ sodium glycolate), which accelerates the nucleophilic attack on the ester carbonyl carbon atoms of the polyester backbone, such as that of PBAT.^22^

In EG-mediated reactions (DP01 and DP07), the *X*_IEC_ values were 73.1 and 69.4%, respectively. However, closer inspection of the detailed distribution species revealed that the Sn­(Bu)_2_O-catalyzed reaction yielded a similar monomer fraction BHET yield. In the 1,4-BD systems (DP04 and DP08), the *X*_IEC_ values were 73.1 and 66.7%, respectively, with DP04 showing a slightly lower HR *M*_w_ (0.80 kDa) compared to DP08 (0.85 kDa). Despite similar results across both diol systems, HR *M*_w_ values were consistently lower under the Sn­(Bu)_2_O catalyst than under NaHCO_3_.

Direct comparison of the glycols remains challenging due to their divergent molar mass distributions, as the degradation products from 1,4-BD are intrinsically larger than their EG counterparts. The discrepancy in glycol comparison is relative to the higher molar masses of the 1,4-BD-derived monomers, bis­(2-hydroxybutyl) terephthalate (310.34 Da) and bis­(2-hydroxybutyl) adipate (290.35 Da), relative to the EG-derived analogues, BHET (254.24 Da) and bis­(2-hydroxyethyl) adipate (234.25 Da), which is reflected in the elevated *M*_n_ and *M*_w_ values observed for the 1,4-BD glycolysis products. Furthermore, the resolution of detailed species differed between systems: in the 1,4-BD reactions, short-chain oligomers could not be resolved from monomers by HR GPC (Figure S5). However, EG-derived products yielded two distinct peaks, enabling clear separation of short-chain oligomers from monomers. However, the X_IEC_ values were similar in the direct comparison among the glycols.

Previous studies have demonstrated that EG exhibits greater reactivity than 1,3-propanediol in the glycolysis of 2-(benzoyloxy)­ethyl benzoate, an effect attributed to the neighboring group interaction between adjacent −OH moieties and the shorter chain length, facilitating transesterification.^23^ Conversely, 1,4-BD has been established as an efficient glycol for PET glycolysis,^24^ and, notably, is itself an original PBAT comonomer. From a structural standpoint, the use of 1,4-BD requires fewer substitution steps to yield symmetric esters, thereby reducing reactivity differences between the glycol medium and the substituted ester products. This structural compatibility between 1,4-BD and the target esters likely explains the comparable glycolysis behavior observed between the two glycols.

The ^1^HNMR spectra presented in Figure S6 show reactions DP01, DP02, and DP03, compared to PBAT. In particular, one can see a decrease in peak intensity at 2.0 ppm and greater spectral complexity in the 3.5–4.5 ppm region. These reactions (conducted at 150, 170, and 190 °C, respectively) were performed in the presence of EG. Unreacted glycol was identified by an intense peak at 3.7 ppm. The formation of EG-based esters is evidenced by new peaks appearing between 3.7–4.0 ppm, which are absent in the original PBAT spectrum. A lower-intensity peak at 3.6 ppm indicates the formation of asymmetric esters with partial EG substitution, specifically, chains in which one terminus remains as a 1,4-BD-based unit, suggesting competitive reactivity between the glycols under these conditions. The chemical shifts near 2.0 ppm are attributed to the methylene protons on the β-carbon of the 1,4-BD structure relative to the ester group. The absence of a corresponding peak in pure EG-based esters confirms that signals in this region are indicative of residual oligomers or partial substitution within the polymer backbone.

^1^HNMR was performed without prior purification to prevent the loss of key compounds through solvent interactions. While this approach preserved the sample’s chemical integrity, it resulted in a more complex interpretation due to sample heterogeneity. To address this, a normalization method was applied in which IP was divided by the sum of the contributions from two specific chemical markers (IAT), as illustrated in Figure 3. The peaks at 3.53 and 3.69 ppm were excluded from the quantitative treatment due to insufficient resolution.

**Figure 3:**
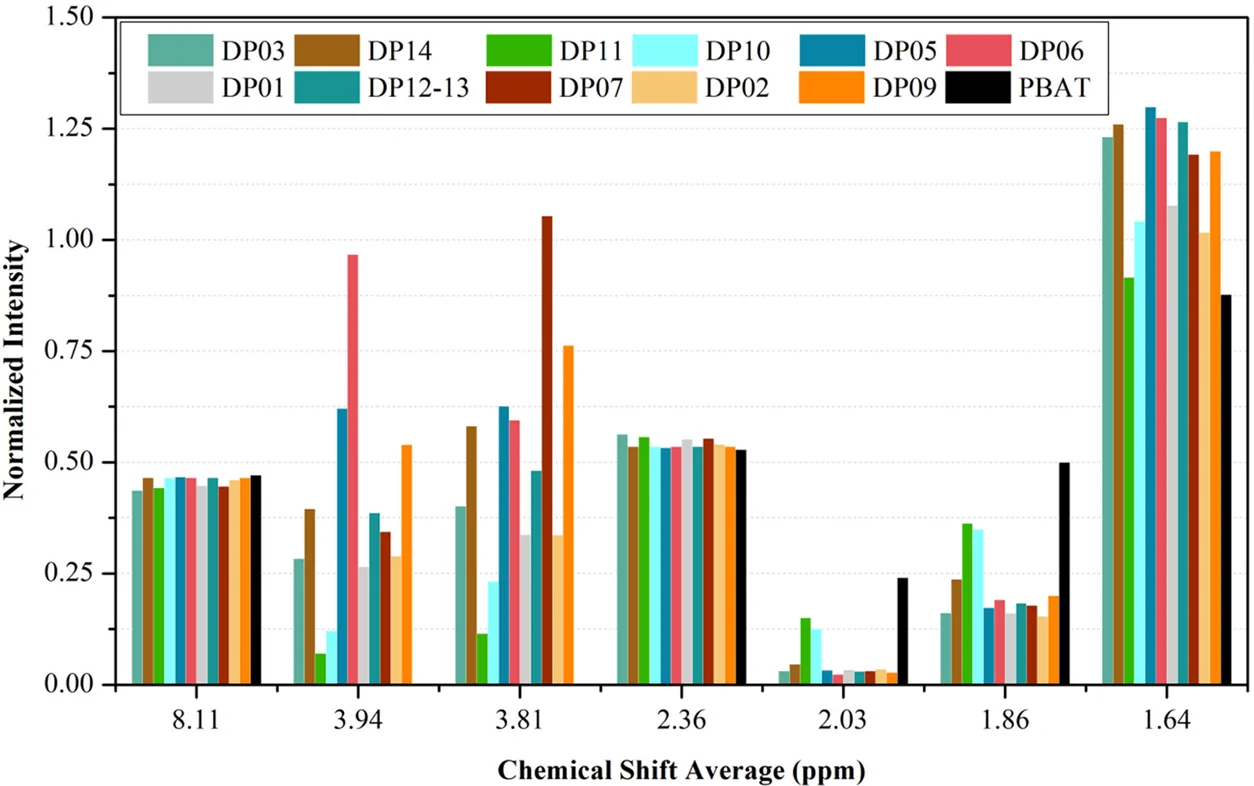
^1^H NMR spectra treatment of the main chemical shifts of the glycolysis products obtained using EG as glycol.

As previously discussed, a decrease in the peak intensity at 2.03 ppm was observed for all of the reactions relative to PBAT. Among these, DP11 exhibited the highest residual intensity in this region, consistent with incomplete conversion under mild reaction conditions; consequently, it served as a baseline for comparison. The signal at 1.64 ppm contains two overlapping contributions: the methylene protons on the internal carbons of the adipate chain and those from free 1,4-BD. While the adipate contribution is expected to remain constant, the intensity in this region typically increases as 1,4-BD is liberated through transesterification, specifically by substituting this glycol with an EG.

Another key signal appears at 1.86 ppm, which is exclusively associated with 1,4-BD-based esters, whether they are fully or partially reacted. The high intensity of these peaks corroborates previous findings, suggesting a high conversion of the polymer into oligomers, alongside a low yield of pure monomers resulting from the complete transesterification of the EG units.

The appearance of peaks at 3.81 and 3.94 ppm confirms the formation of EG-based esters. These peaks were entirely absent in the original PBAT spectrum but were observed in all glycolysis products, including DP11. The higher intensity observed for DP07 at 3.81 ppm suggests a higher frequency of partial EG substitution, indicating a different oligomeric profile. Furthermore, the interaction between the catalyst (NaHCO_3_) and the ester linkages results in Na–O–(C=O)– end groups, which corroborates the formation of nucleophilic alkoxide species. This interaction also explains the relative discrepancy in peak intensities in the sample, where the signal at 3.94 ppm appears less intense than that at 3.81 ppm.

The higher integration values at 3.81 and 3.94 ppm correspond to the low-molar-mass species observed by HR GPC, providing clear evidence of efficient glycolysis when the repolymerization effect is minimized under optimized reaction conditions. Regarding polymer degradation, several combinations of parameters proved to be effective in achieving high glycolysis yields. These include 5 h reactions achieving *X*_IEC_ near 70% and BHET yields higher than 14%.

The ^1^H NMR spectra in Figure S7 illustrate the glycolysis products from reactions with 1,4-BD (DP04 and DP08) in comparison to the original PBAT. Interpretation of these spectra is inherently more complex due to the spectral overlap between the reaction products and residual 1,4-BD, which is visible at 1.63 and 3.53 ppm. A reduction in the peak intensity at 2.0 ppm was observed. In a stoichiometric PBAT repeating unit, six protons are expected in this region, but as glycolysis proceeds, the local concentration of these specific protons decreases. Notably, this reduction is less pronounced when compared with the observed in EG-based glycolysis, likely due to the structural similarity between the glycol and the polymer backbone. The peak at 3.73 ppm is attributed to the formation of hydroxybutyl ester end groups (methylene protons α to the hydroxyl group). However, direct quantitative comparison between DP04 and DP08 was hindered by significant interference from residual 1,4-BD signal.

### Repolymerization Reactions

The polycondensation results are summarized in Table 2. Initially, reactions were proposed using terephthalic and adipic acids as monomers, but the magnetic dispersion of terephthalic acid was hindered during P01, likely because of its low solubility in 1,4-BD and adipic acid. Consequently, subsequent polymerizations were conducted using the DMT and DEA esters along with 1,4-BD as monomers. Adjusting the DMT:DEA molar ratio to 46:54 (P06), which more closely aligns with the composition of commercial PBAT, as determined by ^1^H NMR (Figure S4), yielded slightly higher molar masses in comparison to the 50:50 molar ratio (P07). While the literature suggests that increasing the adipic acid molar ratio beyond 50% can further enhance viscosity and molar masses,^25,26^ the 50:50 ratio was selected for the subsequent repolymerization steps for practical considerations.

**Table 2:** WR GPC Characterization and Polymer Fraction from Polycondensation of Virgin Feedstock (P) and Repolymerization of Glycolyzed Products (RP) and Their Reaction Conditions

		Conditions				WR GPC				^1^HNMR
Sample	Previous Glycolysis	Temp. N_2_ (°C)	Time N_2_ (h)	Temp. vacuum (°C)	Time vacuum (h)	*M*_n_ (kDa)	*M*_w_ (kDa)	*Đ*	Polymer fraction (wt %)	Polymer fraction (wt %)
P01^a^	-	190	3.5	210	3.5	2.7	6.4	2.33	87.4	-
P02^b^	-	190	3.5	210	3.5	2.7	5.2	1.88	93.4	-
P03^b^	-	190	3.5	190	3.5	2.7	4.8	1.78	94.8	-
P04	-	190	3.5	210	3.5	4.2	9.8	2.34	96.5	-
P05	-	190	3.5	190	3.5	4.5	11.4	2.51	97.5	-
P06^c^	-	190	3.0	190	4.0	5.9	12.1	2.05	97.8	-
P07	-	190	3.0	190	4.0	5.5	11.5	2.09	98.0	95.5
RP02	DP04	190	3.0	190	4.0	2.8	5.9	2.12	94.5	90.2
RP03	DP08	190	3.0	190	4.0	1.9	3.0	1.56	90.1	85.2
RP04^d^	DP04	190	3.0	190	4.0	4.3	9.4	2.08	98.0	94.7
RP05^d^	DP15	190	3.0	190	4.0	4.7	10.6	2.12	97.5	95.7

a Terephthalic and adipic acids used as monomers.

b Stoichiometric imbalance of DMT (excess of 30% molar).

c Molar ratio of DMT:DEA of 46:54.

d Repolymerization conducted by total-transfer feeding methodology.

To evaluate the performance of the virgin feedstock polymerization relative to the repolymerization of the glycolysis products (Table 2), the molar mass distributions obtained by W*R* GPC were compared, as illustrated in Figure 4. These comparisons highlight the effects of the glycolysis catalyst type (RP02 vs RP03), the feeding approach (RP02 vs RP04), and the starting glycolysis product (RP04 vs RP05). After glycolysis, Ti­(OBu)_4_ is introduced to catalyze the polycondensation step following previous polycondensation procedures. The advantage of Ti­(OBu)_4_ is that it acts as a powerful transesterification catalyst via a coordination–insertion mechanism,^27^ efficiently driving chain extension and repolymerization to yield high-molar-mass polymer chains while displaying low depolymerization activity.^11^

**Figure 4:**
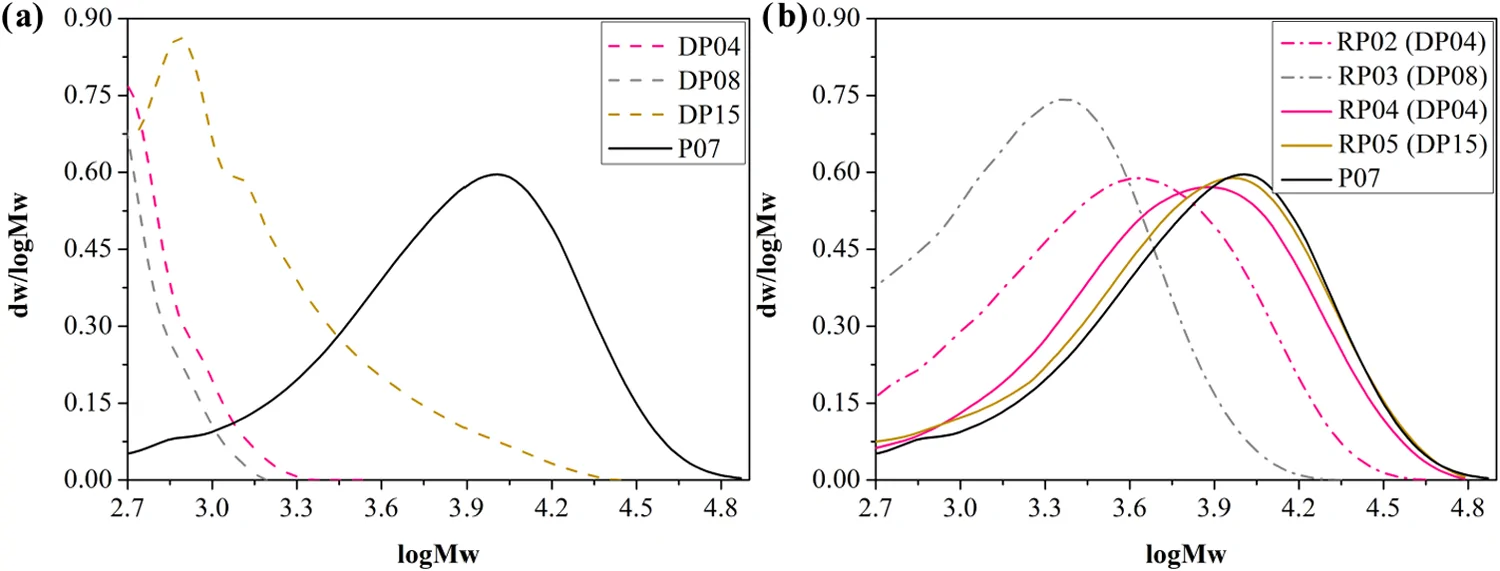
Molar mass distribution obtained by WR GPC, with (a) representing glycolysis products distribution (dash lines) and (b) repolymerization reactions conducted at 190 °C comparing different feeding methods, such as fractional-batch feeding (dash-dot lines) and total-transfer feeding (solid lines).

Comparison of RP02 (DP04) and RP03 (DP08), in which glycolysis products were obtained using Sn­(Bu)_2_O and NaHCO_3_, respectively, with a fractional-batch feeding approach, suggests that using a catalyst like Sn­(Bu)_2_O, which exhibits dual activity in both glycolysis and repolymerization,^11^ leads to higher molar masses in combination with Ti­(OBu)_4_ (Table 2 and Figure 4b). A synergistic effect of the mixed Ti­(OBu)_4_:Sn­(Oct)_2_ catalyst system has been previously observed, maintaining competitive activity while providing greater stability against degradation—due to metal centers with higher coordination numbers in the clusters—relative to the less stable pure compounds.^28^ Conversely, the use of NaHCO_3_ triggers competition between polycondensation and glycolysis reactions, alongside the added Ti­(OBu)_4_, which ultimately reduces the molar masses. This effect could be associated with a known phenomenon in sodium titanate catalysts regarding the formation of oxygen-bound sodium species (such as sodium peroxides), which are catalytically inactive and exhibit higher diffusivity, thereby reducing the number of basic oxygen-containing sites.^29^

Although the molar mass distributions of the repolymerized products did not fully match those of the virgin feedstock (P07), the results clearly demonstrated the potential for successful chemical recycling via repolymerization.

The data suggest that optimizing the homogeneity of the glycolysis product feed is essential to preventing stoichiometric imbalances. A comparison between RP02 and P03 supports the hypothesis that these imbalances were the primary cause of the lower molecular weights in the recycled samples. Specifically, the resulting *M*_n_ values and polymer fractions were remarkably similar, pointing to stoichiometric limitations rather than reaction kinetics as the governing factor.

To mitigate the effects of stoichiometric imbalance, two feeding approaches, fractional-batch and total-transfer feeding, were compared along RP02 and RP04, respectively, with molar mass distributions illustrated in Figure 4b, resulting in a significant increase in both *M*_w_ and polymer fraction without adversely affecting *Đ* (Table 2). These results demonstrate that the feeding approach is critical to enhancing the molar mass of the repolymerized product.

With total-transfer feeding established as the optimal approach, the effect of the starting glycolysis product was evaluated by comparing RP04 (derived from DP04) and RP05 (derived from DP15), which exhibited distinct oligomeric distributions prior to repolymerization (Figure 4a). Despite exhibiting lower overall molar masses than the virgin feedstock (P07) under identical polymerization conditions, both RP04 and RP05 achieved remarkably comparable polymer fractions. Although the glycolysis precursors for RP04 and RP05 were synthesized under contrasting conditions, namely temperature (170 vs 150 °C), reaction times (5 vs 1 h), and catalyst concentrations (0.70 vs 0.35 mol %), the resulting polymers displayed nearly identical molar mass distributions and polymer fraction. This suggests a viable pathway for repolymerization without complex purification steps, which could significantly lower both the environmental footprint and operational costs of the recycling process. Notably, the results for RP05 demonstrate that even low glycolysis conversion can yield polymers with high molar masses, potentially reducing the overall demand for time, energy, and catalyst during the initial depolymerization stage.

In an industrial closed-loop or semi-closed-loop facility, the residual glycol/monomer ratio of the depolymerized stream can fluctuate. Standardizing the formulation by dosing defined amounts of virgin monomers against a precontrolled parameter (1,4-BD content) allows precise adjustment of stoichiometry without requiring complex intermediate purification of the depolymerization mixture. The cleavage of an internal ester linkage by a 1,4-BD molecule consumes one glycol −OH group while simultaneously generating a new hydroxyl end-group on the resulting oligomer/monomeric fragment. Consequently, the total molar concentration of hydroxyl equivalents (−OH) in the reaction mixture remains constant regardless of the extent or degree of conversion of the glycolysis step.

The residual molar fractions of DMT and DEA for each reaction were quantified using ^1^H NMR. Figure S8 highlights the reference peaks utilized to calculate the concentration of these ester monomers, with the resulting values summarized in Table 3. Residual levels of DMT and DEA were estimated by comparing the integrals at 3.94 (DMT methoxy protons) and 1.26 ppm (DEA methyl protons) to the signals at 8.10 and 2.32 ppm, respectively. A higher residual concentration of DEA relative to that of DMT was observed, which is expected as methanol is more volatile and easier to remove from the reaction mass than ethanol. Furthermore, the resulting stoichiometric imbalance among these residual monomers contributes to the lower observed molar mass. It is important to note that the molar residuals may be overestimated by this calculation method, since the integration markers correspond to the alkyl protons of the alcohols, and any oligomeric species with methanol or ethanol end-caps are counted as residual monomers.

**Table 3:** Thermal and Composition Results of Repolymerized Products in Comparison with References (Commercial PBAT and P07)

	DSC					^1^HNMR		
Sample	*T*_m_ (°C)	Δ*H*_m_ (J/g)	*T*_c_ (°C)	Δ*H*_c_ (J/g)	*X*_c_ (%)	Residual DMT (mol %)	Residual DEA (mol %)	T:A Molar Ratio^a^
PBAT	121.3	14.0	38.7	–16.0	12.3	-	-	45.9:54.1
P07	118.7	13.2	76.7	–15.0	12.1	2.54	6.39	50.7:49.3
RP04	132.7	11.1	91.3	–13.2	10.2	3.04	7.51	50.8:49.2
RP05	130.1	11.1	90.6	–12.6	10.3	2.55	6.10	50.4:49.6

a Terephthalate:adipate molar percentage contribution.

Figure 5 illustrates the thermograms of the repolymerized samples (RP04 and RP05) in comparison with both the commercial PBAT baseline and the synthesized control reference (P07). Additionally, Table 3 summarizes the thermal properties determined by DSC analysis. A random distribution of adipate and terephthalate units along the polymer chain results in an intermediate melting temperature near 120 °C, whereas homopolymers based on poly­(butylene adipate) and poly­(butylene terephthalate) exhibit melting points at approximately 60 °C and above 200 °C, respectively.^11,30^ The presence of a broad endothermic peak in all samples within the range of 119 and 132 °C indicated a random sequence distribution of the polymer repeating units, which is corroborated by composition analysis results.

**Figure 5:**
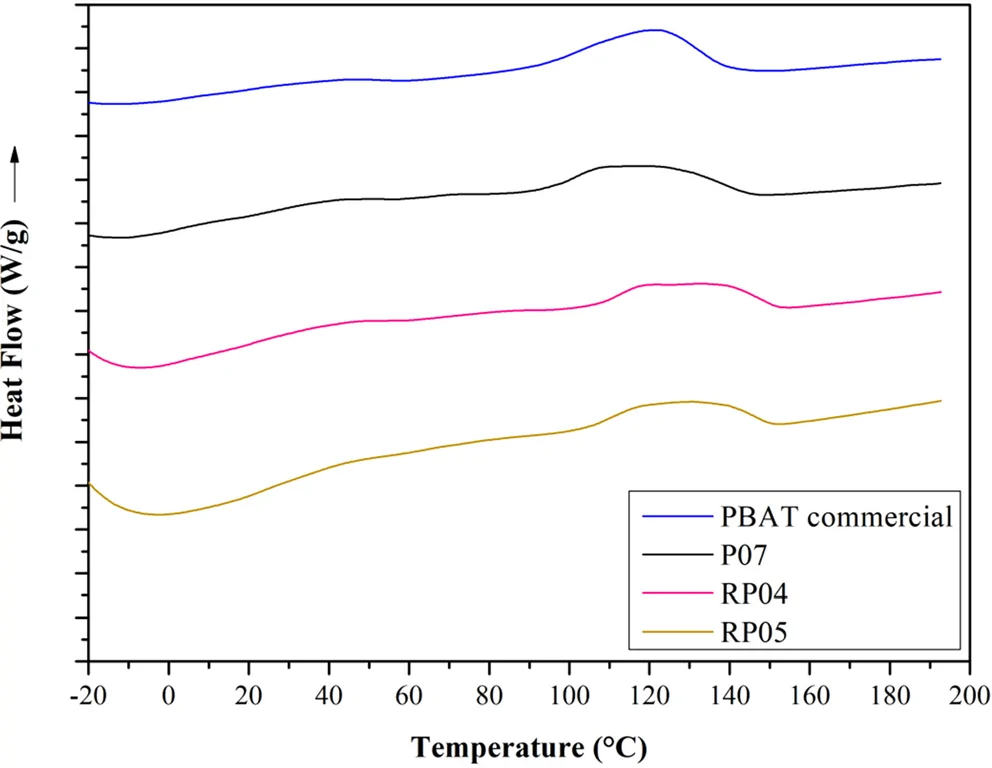
DSC thermograms from second heating scans of the PBAT (commercial and P07) and the repolymerization reaction products (RP04 and RP05).

Additionally, because of their lower molar masses, RP04 and RP05 exhibited higher *T*_c_ values, which may be attributed to the plasticizing effect of residual oligomer chains. This plasticization increases the molecular mobility within the polymer matrix, ultimately reducing crystallinity.^31^ This effect is further corroborated when comparing commercial PBAT and P07, where a lower molar mass also led to an increased *T*_c_.

RP04 and RP05 demonstrate as a chemical proof-of-concept that glycolysis streams can act as a partial, secondary feedstock cofed alongside virgin monomer streams (DMT and DEA) in a PBAT manufacturing context. This supports a gradual transition toward recycled content integration. However, further investigations—such as quantitative elemental analysis to identify the effect of residual metal on polymer composition, alongside property-performance evaluations—are essential for future scale-up and application assessments.

### Life Cycle Impact Assessment (LCIA) Results

Although the repolymerized PBAT obtained in this study has not yet reached the full performance profile of commercial-grade resin, these results establish a laboratory-scale proof of concept for polymer chain reconstruction from crude glycolysis products. Accordingly, the LCA was modeled as a prospective assessment to identify environmental hotspots and challenges prior to industrial scale-up. The LCIA was performed considering 18 impact categories, with their respective category indicators calculated according to the ReCiPe 2016 midpoint approach for each scenario (A, B, and C), as presented in a normalized radar plot (Figure 6).

**Figure 6:**
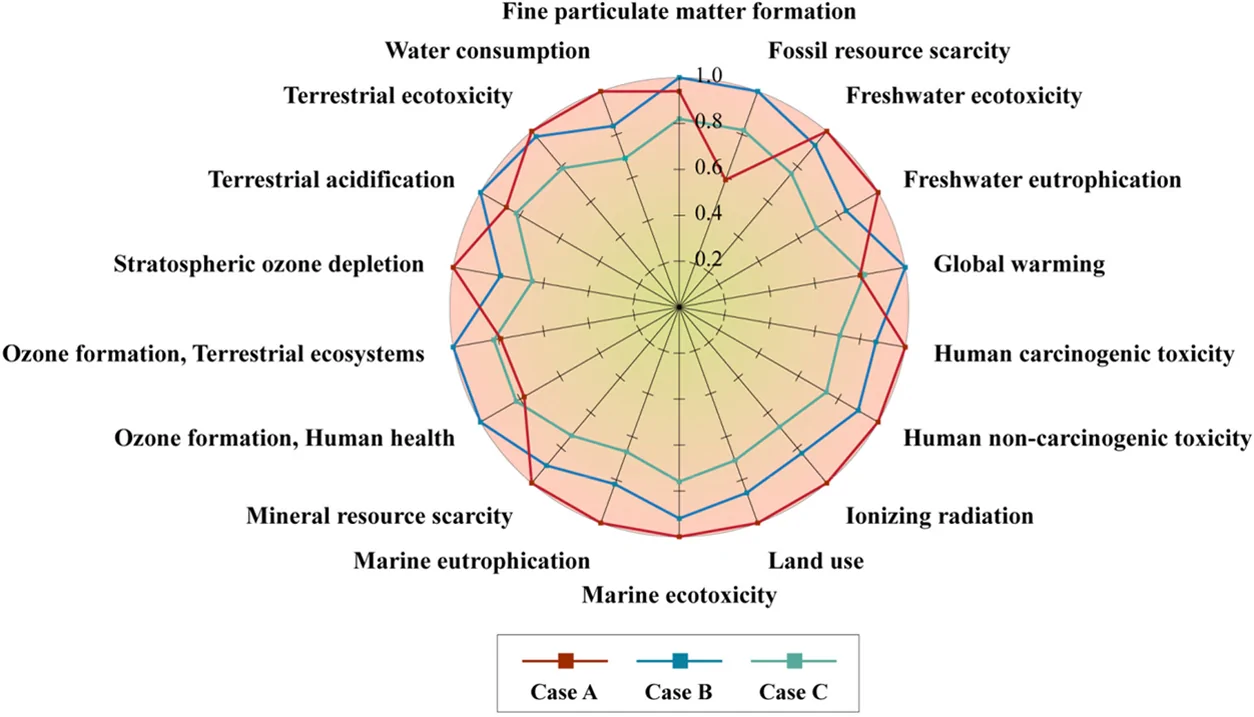
Radar plot shows the normalized category indicators (by higher value in each scenario) of LCIA across Cases A, B, and C.

Case B was modeled representing an environmentally conservative approach to polymer upcycling, as it cumulatively accounts for the impacts of original PBAT production. The comparison between virgin production (Case A) and Case B results in a 6% increase in “particulate matter formation”, a 70% increase in “fossil resource scarcity”, up to a 28% increase in ozone formation parameters (human health and terrestrial ecosystems), a 25% increase in “global warming potential”, and a 14% increase in “terrestrial acidification, while the remaining 12 indicators exhibited reductions ranging from 3% to 21%. Quantitatively, the LCIA results are shown in Figure 7.

**Figure 7:**
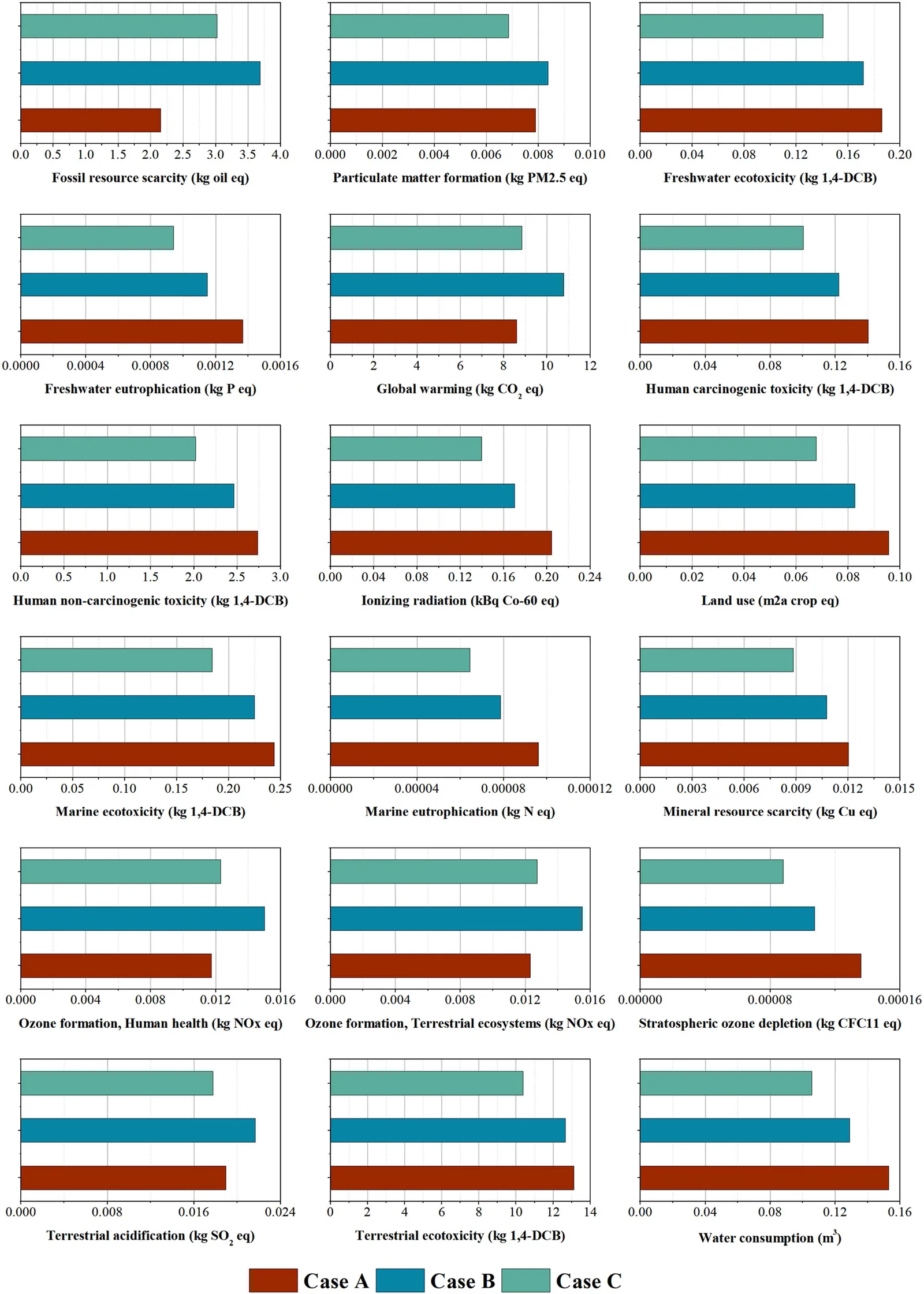
Comparison of quantitative LCIA category indicators across Cases A, B, and C.

Consequently, Case C was modeled using a cutoff approach, effectively setting the environmental burden of the PBAT used (as a waste) in glycolysis to zero. When comparing the virgin production (Case A) with the recycled route (Case C), results indicate that despite a 40% increase in “fossil resource scarcity”, up to a 5% increase in ozone formation parameters (human health and terrestrial ecosystems), and a 3% increase in “global warming potential”, the remaining 14 indicators exhibited significant reductions ranging from 6% to 35%. These results explicitly emphasize the sensitivity of Cases B and C to allocation methods and system boundaries.

The primary factor driving the increase in fossil resource scarcity was the glycolysis stage itself, accounting for 55% of the total impact within the recycled product’s life cycle. This contribution is largely tied to the energy intensity of the process, suggesting that further optimization of heating and reaction times could significantly mitigate this burden. Fossil resource scarcity, ozone formation, and global warming potential are impact categories closely linked to fossil fuel consumption and combustion byproducts.

These cases were modeled on the basis of a fossil-intensive energy matrix to reflect standard global industrial conditions. Beyond the boundaries of the present model, the integration of glycolysis into existing polymer plants offers prospective opportunities to reduce overall resource demand by the application of flow integration, heat recovery condensing units, and regenerative heat exchangers. Furthermore, transitioning to a renewable energy matrix represents a prospective scenario that could further mitigate environmental impacts, providing a relevant pathway for future eco-design and scale assessments.

As another prospective recommendation for future process optimization, reducing the stoichiometric excess of 1,4-BD presents a promising direction for further refinement. Hypothetically, decreasing the 1,4-BD input would increase the mass fraction of zero-impact waste material relative to virgin feedstock, which is anticipated to enhance both the stoichiometry balance for repolymerization and the overall environmental profile. Additionally, sourcing 1,4-BD from sustainable feedstocks (such as lignocellulosic biomass, which the literature indicates as environmentally favorable compared to fossil-based counterparts)^32–34^ represents an attractive route for future LCA scenario modeling.

While PBAT is classified as a biodegradable polymer, its petrochemical origin results in a significantly higher carbon footprint compared to biobased alternatives.^13^ Furthermore, an appropriate end-of-life scenario via industrial composting requires specialized infrastructure to prevent anaerobic degradation, since without such controls, the process can release methane, a greenhouse gas with a global warming potential significantly higher than that of carbon dioxide.^35,36^ Consequently, mitigating these environmental impacts through chemical recycling, such as glycolysis, offers a promising pathway under specific system boundaries to enhance the sustainability profile of this polymer by promoting a circular economy.

It is important to emphasize that this prospective LCA serves as a screening assessment to identify environmental hot spots and guide process optimization prior to scaling. To contextualize how environmental metrics may evolve under mature industrial conditions, two primary factors will heavily influence the system’s life cycle performance: energy intensity and feedstock variability. Industrially integrated processes typically benefit from economies of scale and heat integration, which substantially lower thermal energy inputs per unit mass compared with lab-scale setups. Conversely, postconsumer PBAT streams inevitably introduce variability in quality and contaminants, which may require additional pretreatment steps or stoichiometric adjustments. Accordingly, incorporating industrial energy modeling and empirical data on postconsumer waste variability represents a key priority for future process upscaling. Furthermore, given that the glycolysis inventory incorporates literature-derived data and is sensitive to allocation choices and system boundaries, these results provide preliminary indications of potential environmental advantages rather than definitive proof of commercial viability.

## Conclusion

This work demonstrates PBAT glycolysis as a highly efficient and versatile chemical recycling pathway across a broad range of conditions, achieving internal ester bond conversions (*X*_IEC_) exceeding 70% and oligomers with HR *M*_w_ of 0.6–1.4 kDa. While the molar masses achieved through the repolymerization of glycolysis products (*M*_w_ up to 10.6 kDa) were lower than those from virgin feedstock (11.2 kDa), the results suggest that reconstruction of the polymer chain is possible by this method. Direct repolymerization without complex intermediate purification was demonstrated as a chemically viable route at the laboratory scale. Comparative LCA data applied to a hypothetical scale-up revealed that applying a zero-burden cutoff approach (Case C) yielded environmental impact reductions of up to 35% across key indicators compared to virgin PBAT production (Case A). Overall, these findings position the integration of crude glycolysis products into polymerization streams as a promising laboratory-scale proof-of-concept for PBAT circularity. The accompanying screening LCA highlights key environmental hotspots—particularly energy duty during depolymerization—offering valuable guidelines for optimizing continuous closed-loop operations. Key process limitations—specifically the stoichiometric tuning of crude feeds, the low molar masses of repolymerized products, and the role of native plastic additives or additive-catalyst interactions—represent critical avenues for future process optimization, scale-up assessments, and dedicated techno-economic analyses. Other challenges to implement this approach are related to real waste streams (e.g., starch blends) and contaminated postconsumer feedstock.

## Supplementary Material



## Notes

Additional experimental/methodology details, characterization data such as ^1^HNMR and GPC distribution, and LCA detailed information: Romera_PBAT_De-Repolymerization_Supporting_Information (PDF)

## Abbreviations

Δ*H*_m_: melting enthalpy of the sample
Δ*H*_m_°: melting enthalpy of the 100% crystalline PBAT
1,4-BD: 1,4-butanediol
^1^HNMR: proton nuclear magnetic resonance
BHET: bis­(2-hydroxyethyl) terephthalate
Đ: dispersity
DEA: diethyl adipate
DMT: dimethyl terephthalate
d*n*/d*c*: refractive index increment
DSC: differential scanning calorimetry
EG: ethylene glycol
*f*: corrected refractive factor
GPC: gel permeation chromatography
HPLC: high-performance liquid chromatography
HR GPC: high-resolution gel permeation chromatography
IP: integration peak (IP)
IAT: integration of the sum of adipate and terephthalate peaks
LCA: life cycle assessment
LCIA: life cycle impact assessment
*M*_n_: number-average molar mass, *M*_w_, weight-average molar mass
NaHCO_3_: sodium bicarbonate
PBAT: poly­(butylene adipate-*co*-terephthalate)
SCF: subcalibration fraction
Sn­(Bu)_2_O: dibutyltin­(IV) oxide
*T*_c_: crystallization temperature
Ti­(OBu)_4_: titanium­(IV) butoxide
*T*_m_: melting temperature
W*R* GPC: wide-range gel permeation chromatography
*X*_c_: percent crystallinity
X_IEC_: conversion of internal ester chains.

## References

[1] Organisation for Economic Co-operation and Development (OECD) . Waste Management and the Circular Economy in Selected OECD Countries: Evidence from Environmental Performance Reviews. In OECD Environmental Performance Reviews; OECD Publishing, 2019.

[2] Organisation for Economic Co-operation and Development (OECD) . Plastic Pollution Is Growing Relentlessly As Waste Management And Recycling Fall Short, Says OECD; OECD. https://www.oecd.org/en/about/news/press-releases/2022/02/plastic-pollution-is-growing-relentlessly-as-waste-management-and-recycling-fall-short.html. (published February 22, 2022).

[3] Schyns Z. O. G., Shaver M. P. (2021). Mechanical Recycling of Packaging Plastics: A Review. Macromol. Rapid Commun..

[4] Lamberti F. M., Román-Ramírez L. A., Wood J. (2020). Recycling of Bioplastics:
Routes and Benefits. J. Polym. Environ..

[5] Debowski M., Iuliano A., Plichta A., Kowalczyk S., Florjanczyk Z. (2019). Chemical recycling of polyesters. Polimery.

[6] Clark R. A., Shaver M. P. (2024). Depolymerization within a Circular
Plastics System. Chem. Rev..

[7] Jian J., Xiangbin Z., Xianbo H. (2020). An overview
on synthesis, properties
and applications of poly­(butylene-adipate-co-terephthalate)–PBAT. Adv. Ind. Eng. Polym. Res..

[8] Ismail M., Abouhmad A., Warlin N., Pyo S.-H., Örn O. E., Al-Rudainy B., Tullberg C., Zhang B., Hatti-Kaul R. (2024). Closing the
loop for poly­(butylene-adipate-co-terephthalate) recycling: depolymerization,
monomers separation, and upcycling. Green Chem..

[9] Wu W., Zhai H., Wu K., Wang X., Rao W., Ding J., Yu L. (2024). Cheap organocatalyst
diphenyl phosphate
for efficient chemical recycling of poly­(lactic acid), other polyesters
and polycarbonates. Chem. Eng. J..

[10] Pang W., Li B., Wu Y., Zeng Q., Yang J., Zhang Y., Tian S. (2024). Upgraded recycling
of biodegradable PBAT plastic: Efficient hydrolysis
and electrocatalytic conversion. Chem. Eng.
J..

[11] Parodi A., Arpaia V., Samorì C., Mazzocchetti L., Galletti P. (2023). Novel Strategies for Recycling Poly­(butylene
adipate-co-terephthalate)-Starch-Based
Plastics: Selective Solubilization and Depolymerization–Repolymerization
Processes. ACS Sustainable Chem. Eng..

[12] Iturrondobeitia M., Alonso L., Lizundia E. (2023). Prospective
life cycle assessment
of poly (ethylene terephthalate) upcycling via chemoselective depolymerization. Resour. Conserv. Recycl..

[13] Merchan A. L., Fischöder T., Hee J., Lehnertz M. S., Osterthun O., Pielsticker S., Schleier J., Tiso T., Blank L. M., Klankermayer J. (2022). Chemical Recycling of Bioplastics: Technical
Opportunities to Preserve Chemical Functionality as Path towards a
Circular Economy. Green Chem..

[14] Surendren A., Mekonnen T. H. (2026). Reactive Chain Extension
and Nanoclay-Enabled Foaming
of High-MFI PBAT for High-Expansion, Compostable Polymer Foams. ACS Sustainable Chem. Eng..

[15] Luo C., Zhou Y., Chen Z., Bian X., Chen N., Li J., Wu Y., Yang Z. (2024). Comparative life cycle assessment
of PBAT from fossil-based and second-generation generation bio-based
feedstocks. Sci. Total Environ..

[16] Turk J., Cotič Z., Mladenovič A., Šajna A. (2015). Environmental
Evaluation of Green Concretes versus Conventional Concrete by Means
of LCA. Waste Manag..

[17] Huglin, M. B. Light Scattering from Polymer Solutions; Academic Press: London, 1972.

[18] Hanley B. F. (2020). A Practical
Method for Estimating Specific Refractive Index Increments for Flexible
Non-Electrolyte Polymers and Copolymers in Pure and Mixed Solvents
Using the Gladstone-Dale and Lorentz-Lorenz Equations in Conjunction
with Molar Refraction Structural Constants, and Solvent Physical Property
Databases. Mater. Today Commun..

[19] Striegel, A. M. ; Yau, W. W. ; Kirkland, J. J. ; Bly, D. D. Modern Size-Exclusion Liquid Chromatography: Practice of Gel Permeation and Gel Filtration Chromatography, 2nd ed.; John Wiley & Sons: Hoboken, NJ, 2009.

[20] Hobbs L. A., Smith P. J. (1992). Mono-organotin­(IV)
compounds as esterification and
transesterification catalysts. Appl. Organomet.
Chem..

[21] Shukla S. R., Kulkarni K. S. (2002). Depolymerization of Poly­(Ethylene Terephthalate) Waste. J. Appl. Polym. Sci..

[22] Otera J. (1993). Transesterification. Chem. Rev..

[23] Kumar P., Sumpter B. G., Saito T., Davis R. J. (2024). Importance of hydrogen
bonding in base-catalyzed transesterification reactions with vicinal
diols. J. Catal..

[24] Mansour S. H., Ikladious N. E. (2002). Depolymerization
of poly­(ethylene terephthalate) wastes
using 1,4-butanediol and triethylene glycol. Polym. Test..

[25] Herrera R., Franco L., Rodríguez-Galán A., Puiggalí J. (2002). Characterization and Degradation Behavior of Poly­(butylene
adipate-co-terephthalate)­s. J. Polym. Sci. A:
Polym. Chem..

[26] Wang H.-T., Wang J.-M., Wu T.-M. (2019). Synthesis
and characterization of
biodegradable aliphatic–aromatic nanocomposites fabricated
using maleic acid-grafted poly­[(butylene adipate)-co-terephthalate]
and organically modified layered zinc phenylphosphonate. Polym. Int..

[27] Guan Z., Zhang J., Zhou W., Zhu Y., Liu Z., Zhang Y., Zhang Y. (2023). Mechanistic Details of the Titanium-Mediated
Polycondensation Reaction of Polyesters: A DFT Study. Catalysts.

[28] Jansen J. H., Powell A. B., Specht S. E., Gerislioglu S., Hermans I. (2022). Understanding the Structure and Reactivity of Mixed
Titanium­(IV) Alkoxide and Tin­(II)/(IV) Carboxylates as Esterification
Catalysts. ACS Sustainable Chem. Eng..

[29] Marciniuk L. L., Hammer P., Pastore H. O., Schuchardt U., Cardoso D. (2014). Sodium titanate as basic catalyst
in transesterification
reactions. Fuel.

[30] Cranston E., Kawada J., Raymond S., Morin F. G., Marchessault R. H. (2003). Cocrystallization
Model for Synthetic Biodegradable Poly­(butylene adipate-co-butylene
terephthalate). Biomacromolecules.

[31] Olivas
Alonso C., Chiralt A., Torres-Giner S. (2026). Incorporation
of Fully Bio-Based Butylene Succinate Oligomers into Poly­(butylene
succinate) by Melt Mixing. Polymers.

[32] Guo H., Liu H., Jin Y., Zhang R., Yu Y., Deng L., Wang F. (2022). Advances in
Research on the Bio-Production of 1,4-Butanediol by the
Engineered Microbes. Biochem. Eng. J..

[33] Zhang K., Zhao W., Xu L., Li Y. (2026). Advances in Bio-Based
Production of 1,4-Butanediol. Processes.

[34] Forte A., Zucaro A., Basosi R., Fierro A. (2016). LCA of 1,4-Butanediol
Produced via Direct Fermentation of Sugars from Wheat Straw Feedstock
within a Territorial Biorefinery. Materials.

[35] Changwichan K., Silalertruksa T., Gheewala S. H. (2018). Eco-efficiency assessment of bioplastics
production systems and end-of-life options. Sustainability.

[36] Akbarian-Saravi N., Larizadeh R., Gupta A., Shum D., Milani A. S. (2025). Life Cycle
Assessment Sheds New Insights Toward Effective Sustainable Management
of Biodegradable Resin Blends Used in Packaging: A Case Study on PBAT. Sustainability.

