# Supplementary material for: Chemical Recycling of Poly(butylene adipate-co-terephthalate): A Glycolysis–Repolymerization Approach
Source: ACS Omega. 2026 Oct 21;11(43):67892–905. doi: 10.1021/acsomega.6c10047 (PMC13736923; doi:10.1021/acsomega.6c10047)
Supplement: Supplementary file 1 [file ao6c10047_si_001.pdf]

SUPPORTING INFORMATION

Chemical Recycling of Poly(butylene adipate-co-terephthalate): A Glycolysis−Repolymerization Approach

Cristian de Oliveira Romera[1], Alessandra da Rocha Duailibe Monteiro[2], Bruno Francisco Oechsler[1], Claudia Sayer[1], Caio Gomide Otoni[3,4], Pedro Henrique Hermes de Araújo[1*]

[1]Federal University of Santa Catarina, Campus Universitário Reitor João David Ferreira Lima, Florianópolis, 88040-900, Santa Catarina, Brazil.

[2]Fluminense Federal University, Rua Passos da Pátria, n.156 bloco D sala 305, Niterói, 24210-240, Rio de Janeiro, Brazil.

[3]University of Campinas (Unicamp), Institute of Chemistry, Rua Monteiro Lobato, 270, Campinas, 13083-862, São Paulo, Brazil.

[4]Graduate Program in Materials Science and Engineering (PPGCEM), Federal University of São Carlos (UFSCar), Rod. Washington Luís, km 235, São Carlos, 13565-905, São Paulo, Brazil.

[*]E-mail: pedro.h.araujo@ufsc.br

Experimental Section

Materials

Table S1. Technical datasheet properties and additional information of commercial-grade PBAT (grade MP0345; lot no. 107-24/007).

Property	Method	Test Condition	Typical Values	Unit
Melt Flow Rate (MFR)	ISO 1133	190 °C, 2,16 kg	2.0-5.0	g/10 min
Specific gravity	ISO 1183	23 °C	1.22	g/cm^3
Melting point	DSC	10 °C/min	115-125	°C
Tensile Strength	ISO 527	50 mm/min	20-30	MPa
Elongation	ISO 527	50 mm/min	>600	%
Manufacturing date: August 2024; Expiration date: August 2026.				
**Storage recommendation:** Temperatures during storage may not exceed 60 ˚C at any time. Storage time in an unopened bag may not surpass 12 month at room temperature (23 ˚C).				

Glycolysis Reactions

Table S2. Formulation data and reactant quantities for the synthesized glycolyzed products.

Reaction	m_{PBAT} (g)	m_{glycol} (g)	Glycol Type	$m_{catalyst}$ (g)	Catalyst Type
DP01	5.0000	5.9112	EG	0.0415	$Sn(Bu)_2O$
DP02	5.0000	5.9112	EG	0.0415	$Sn(Bu)_2O$
DP03	5.0000	5.9112	EG	0.0415	$Sn(Bu)_2O$
DP04	5.0000	8.5830	1,4-BD	0.0415	$Sn(Bu)_2O$
DP05	5.0000	5.9112	EG	0.0830	$Sn(Bu)_2O$
DP06	5.0000	5.9112	EG	0.0415	$Sn(Bu)_2O$
DP07	5.0000	5.9112	EG	0.1000	$NaHCO_3$
DP08	5.0000	8.5830	1,4-BD	0.1000	$NaHCO_3$
DP09	5.0000	5.9112	EG	0.0089	$Sn(Bu)_2O$
DP10	5.0000	5.9112	EG	0.0089	$Sn(Bu)_2O$
DP11	5.0000	5.9112	EG	0.0207	$Sn(Bu)_2O$
DP12	5.0000	5.9112	EG	0.0252	$Sn(Bu)_2O$
DP13	5.0000	5.9112	EG	0.0252	$Sn(Bu)_2O$
DP14	5.0000	5.9112	EG	0.0207	$Sn(Bu)_2O$
DP15	5.0000	8.5830	1,4-BD	0.0207	$Sn(Bu)_2O$

(Re)Polymerization Reactions

Table S3. Formulation data and reactant quantities for the polymerization reactions.

Reaction	m_{DMT} (g)	m_{TA}[a] (g)	m_{DEA}[b] (g)	m_{AA} (g)	$m_{1,4\text{-}BD}$[c] (g)	$m_{Ti(OBu)_4}$ (g)
P01	-	4.0000	-	3.5187	4.5568	0.0672
P02	4.0000	-	3.1820	-	2.9776	0.0439
P03	4.0000	-	3.1820	-	2.9776	0.0439
P04	4.0000	-	4.1660	-	3.8983	0.0575
P05	4.0000	-	4.1660	-	3.8983	0.0575
P06	4.0000	-	4.9074	-	4.4611	0.0641

P07	4.0000	-	4.1660	-	3.8983	0.0575

[a]TA is terephthalic acid; b) AA is adipic acid; and c) 2.1 mol related to 1 mol of DMT (or DEA).

Table S4. Formulation data and reactant quantities for the repolymerization reactions.

**Reaction**	**Glycolysis Based**	$m_{Glycolysis}$ **(g)**	m_{DMT} **(g)**	m_{DEA} **(g)**	$m_{Ti(OBu)_4}$ **(g)**
RP02[a]	DP04	4.0000[a]	2.5634	2.6698	0.0368
RP03[a]	DP08	4.0000[a]	2.5634	2.6698	0.0368
RP04[b]	DP04	13.6245[b]	8.8069	9.1723	0.1266
RP05[b]	DP15	13.6037[b]	8.8091	9.1746	0.1266

[a]The stoichiometry is calculated based on fractional amount present (fractional-batch feeding) of 1,4-BD, considering 2.1 mol of glycol related to 1 mol of DMT (or DEA); [b]Total mass introduced (total-transfer feeding) was used and stoichiometric calculation was based on 1,4-BD added (8.5830 g), also considering 2.1 mol of 1,4-BD related to 1 mol of (or DEA).

Characterization

Wide range (WR) molar mass distribution, polymer fraction and sub-calibration fraction (SCF): The sub-calibration fraction (SCF) obtained from WR GPC was used to quantify the fraction of lower-molar-mass species below 580 Da. WR M_n and M_w were derived exclusively from the integrated chromatogram area (WR GPC integration area) at or above 580 Da.

Figure S1. Integration cutoff methodology used to evaluate wide range (WR) molar masses above 580 Da, corresponding to the lowest available WR GPC calibration standard, and SCF for distribution below 580 Da.

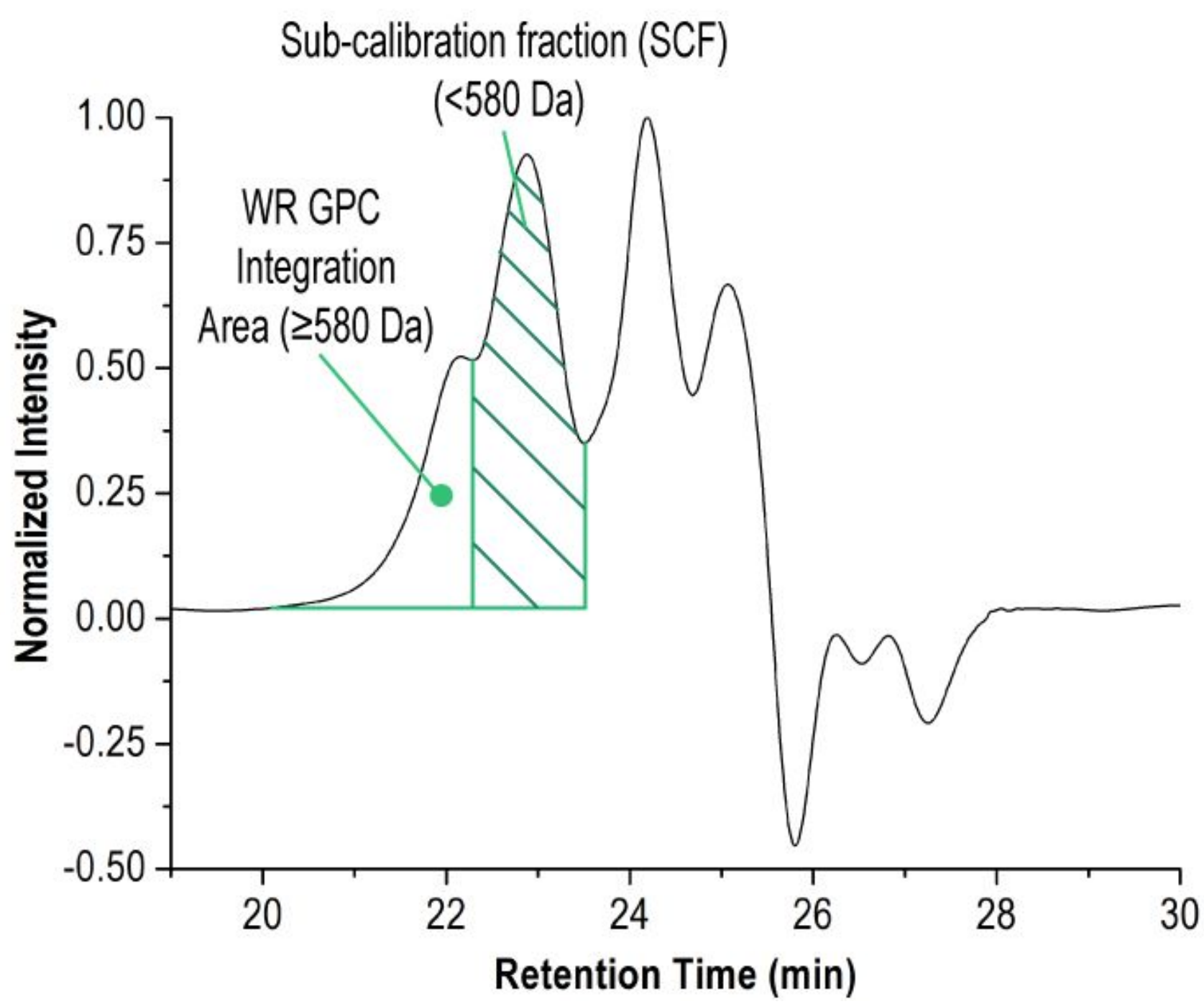

SCF was applied as qualitative measurement in order to select samples for HR GPC, protecting the column from higher molar mass fraction. Table S5 shows the summary of the values obtained from this approach.

Table S5. SCF values of the synthesized glycolyzed products from WR GPC.

Reaction	SCF (%)
DP01	69.4
DP02	65.8
DP03	75.8
DP04	70.1
DP05	79.6
DP06	82.0
DP07	80.0
DP08	75.4
DP09	73.0
DP10	39.5
DP11	26.1
DP12	65.6
DP13	76.3
DP14	69.4
DP15	34.8

The PBAT/polymer fraction was determined by integrating the WR GPC chromatogram from a molecular weight cutoff of 3,370 Da (PS standard) toward higher molar masses. This integrated area was then normalized against the total area under the curve of the WR GPC chromatogram to calculate the relative polymer content.

High resolution (HR) low molar mass distribution for oligomeric species:

Figure S2. The molar mass distribution of the HR GPC standards. DMI is dimethyl itaconate and PS is polystyrene.

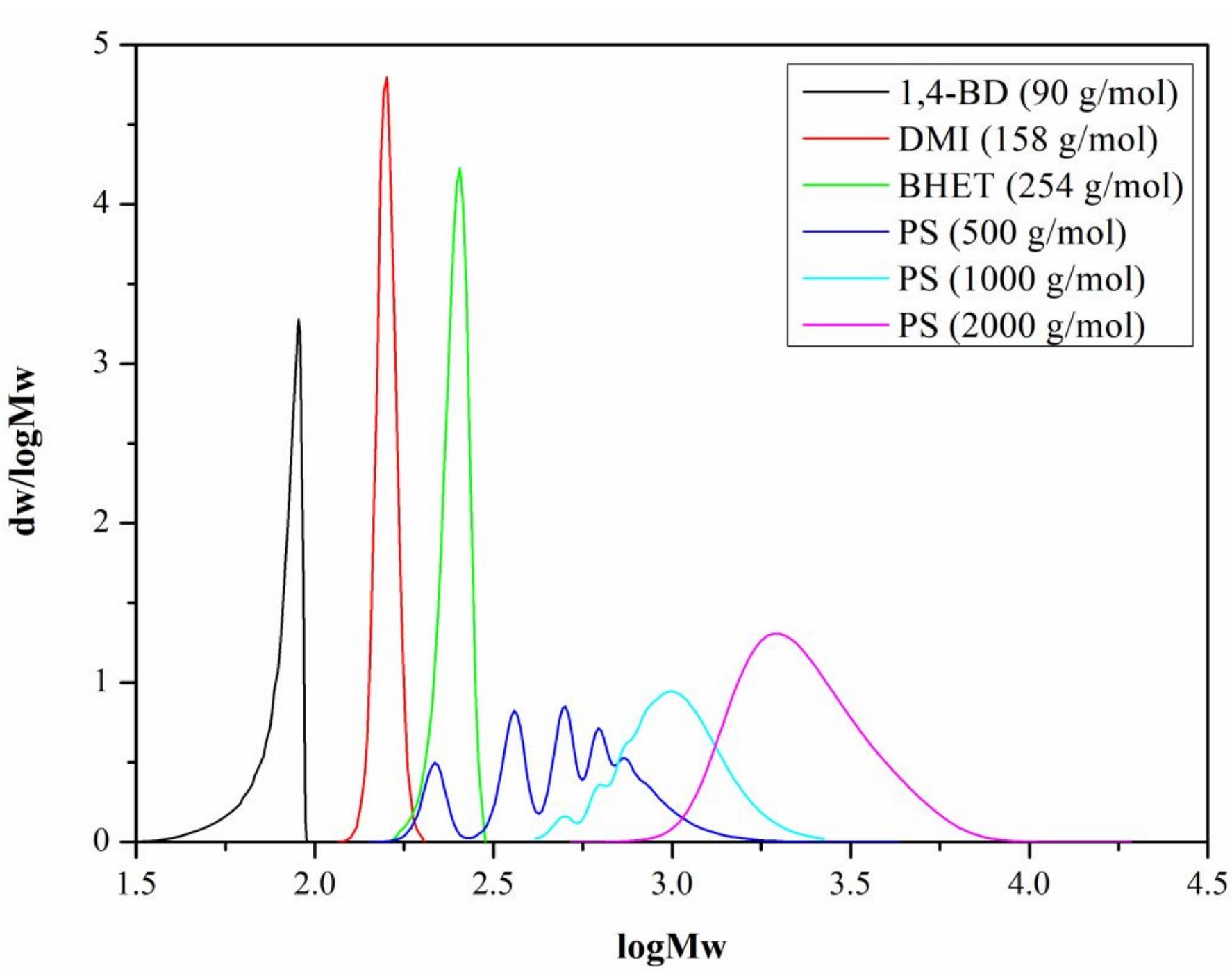

For the mass quantification of the oligomeric fraction via HR GPC, a correction based on molar refractivity (R_m) as described by the Lorentz-Lorenz equation (Eq. S1) was applied to adjust the detector signal. This correction accounts for the refractive index (n, equation S2) and the difference between the refractive indices of analyte and solvent (Δn, Eq. S3) to prevent errors in peak area integration regarding mass concentration, where M is the molecular weight, ρ is the density of the species, and $n_{chloroform}$ is assumed to be 1.445.

$$R_m = \frac{n^2 - 1}{n^2 + 2} \cdot \frac{M}{\rho} \quad \text{(S1)}$$

$$n = \sqrt{\frac{M + 2 \cdot \rho \cdot R_m}{M - \rho \cdot R_m}} \quad \text{(S2)}$$

$$\Delta \text{n} = n - n_{\text{chloroform}} \quad \text{(S3)}$$

The parameters and the calculation of the corrected refractive factor (f) are presented in Table S6.

Table S6. Parameters adopted in refractive index correction.

Parameter	R_m (cm^3)	n	M (g/mol)	ρ (g/cm3)	Δn	f
BHET	61.5	1.500	254.24	1.22	0.055	2.16
BHET dimer	107.3	1.518	446.41	1.26	0.073	1.64
BHET trimer	153.1	1.531	638.58	1.29	0.086	1.39
BHET tetramer	200.3	1.544	830.75	1.31	0.099	1.20
BHET pentamer	250.19	1.564	1022.92	1.33	0.119	1.00
BHEA	56.2	1.450	234.25	1.12	0.005	24.17
BHEA dimer	96.7	1.455	406.42	1.14	0.010	12.10
BHEA trimer	137.2	1.458	578.6	1.15	0.013	9.41
BHEA tetramer	177.5	1.456	750.78	1.15	0.011	10.74
BHEA pentamer	217.8	1.459	923.56	1.16	0.014	8.31

The obtained corrected refractive factors were applied as an average for each molar mass range from the GPC integration, considering a terephthalate : adipate composition of 47:53 %wt calculated from the ^{1}HNMR of commercial PBAT.

$$f_{\text{monomers}} = 0.47 \cdot f_{\text{BHET}} + 0.53 \cdot f_{\text{BHEA}} = 7.43 \quad \text{(S4)}$$

$$f_{\text{dimers}} = 0.47 \cdot f_{\text{BHET dimer}} + 0.53 \cdot f_{\text{BHEA dimer}} = 7.19 \quad \text{(S5)}$$

$$f_{\text{oligomers}} = 0.47 \cdot \left(f_{\text{BHET trimer}} + f_{\text{BHET tetramer}} + f_{\text{BHET pentamer}} \right) + 0.53 \cdot \left(f_{\text{BHEA trimer}} + f_{\text{BHEA tetramer}} + f_{\text{BHEA pentamer}} \right) = 5.59 \quad \text{(S6)}$$

The corrected refractive factors were multiplied by the GPC integration area of each oligomeric fraction to obtain the mass fraction of species. The corrected areas are presented in Table S7, while the estimated mass fraction of monomers and oligomers are presented in Table S8.

Table S7. Correction of HR GPC areas based on corrected refractive factors.

Sample	Long-chain oligomers area	Long-chain oligomers area corrected	Short-chain oligomers (dimers) area	Short-chain oligomers (dimers) area corrected	Monomers area	Monomers area corrected	Total Area corrected
DP03	1271710	7109110	1192876	8572655	664691	4938877	14783242
DP14	1705761	9535541	1598055	11484491	642768	4775982	17966234
DP10	2594691	14504836	819993	5892915	146322	1087222	9574828
DP05	1573887	8798339	1418754	10195937	838440	6229890	17999714
DP06	1462151	8173713	1389879	9988425	803395	5969494	17420071
DP01	1773188	9912471	1481987	10650363	992680	7375945	19799497
DP12	1027704	5745069	1071624	7701272	661282	4913547	13642523
DP13	1185458	6626945	1259056	9048260	774197	5752543	15986262
DP07	1637217	9152367	1432761	10296599	826840	6143699	18077514
DP02	1646829	9206100	1306107	9386394	973480	7233283	18266506
DP09	1271710	7109110	1192876	8572655	664691	4938877	14783242

Table S8. Estimative of mass fraction of monomers and oligomers based of correction of HR GPC areas.

Sample	Long-chain oligomers (wt%)	Short-chain oligomers/dimers (wt%)	Monomers (wt%)
DP03	8.6	58.0	33.4
DP14	9.5	63.9	26.6
DP10	27.1	61.5	11.4

DP05	8.7	56.6	34.6
DP06	8.4	57.3	34.3
DP01	9.0	53.8	37.3
DP12	7.5	56.5	36.0
DP13	7.4	56.6	36.0
DP07	9.1	57.0	34.0
DP02	9.0	51.4	39.6
DP09	8.6	58.0	33.4

BHET Yield: The calculation of BHET yield from PBAT glycolysis was based on the stoichiometry of single PBAT repeating unit of 420 $gmol^{-1}$. According to ^{1}HNMR of the commercial PBAT, each unit theorically yields 0.9 mol of BHET and 1.1 mol of bis(2-hydroxyethyl) adipate (BHEA). The following equations were employed to estimate the final BHET yield:

$$C_{100\%} = \left[\left(\frac{m_{\text{PBAT}}}{0.9 \times 420\ \text{g}\cdot\text{mol}^{-1}} \times 254.54\ \text{g}\cdot\text{mol}^{-1}\right)\frac{1}{V_{\text{Free EG+1,4-BD}}}\right]\left(\frac{V_{\text{sample}}}{V_{\text{methanol}}}\right) \quad \text{(S7)}$$

$$V_{\text{sample}} \approx \frac{m_{\text{sample}}\left(m_{\text{PBAT}} + m_{\text{EG}}\right)}{\left(m_{\text{PBAT}} \times \rho_{\text{PBAT}}\right) + \left(m_{\text{EG}} \times \rho_{\text{EG}}\right)} \quad \text{(S8)}$$

$$\text{BHET Yield} = \frac{C_{sample}}{C_{100\%}} \times 100 \quad \text{(S9)}$$

EG-glycolysis normalization procedure (^{1}H NMR): For glycolysis products, peak integration was used to assess the ester distribution. Each individual peak integration (IP) at 1.64, 1.86, 2.03, 2.36, 3.81, 3.94, and 8.11 ppm were normalized to the sum of adipate and terephthalate contributions (IAT), taken from the characteristic signals at 2.36 and 8.11 ppm, respectively. The following equations were applied considering x as the evaluated chemical shift average, and n as the sample number:

$$IAT_n = IP_{2.36,n} + IP_{8.11,n} \quad \text{(S10)}$$

$$\text{Normalized Intensity}_{x,n} = \frac{IP_{x,n}}{IAT_n} \tag{S11}$$

^{1}H NMR for Residual of DMT and DEA calculation: The calculation of DMT yields and residuals from polymerization reactions was based on the relation with of overall terephthalate unit represented by integration value at 8.09 ppm ($I_{8.10}$), which represents 4 hydrogens, minus the residual of DMT marker at 3.94 ppm ($I_{3.94}$), representing 6 hydrogens. The following equations were employed to estimate the final yield and residual of DMT, respectively:

$$Yield_{\mathrm{DMT}} = 100 \cdot \left\{ I_{8.10} - \left[\left(4 \text{ hydrogens} \right) \cdot \left(\frac{I_{3.94}}{6 \text{ hydrogens}} \right) \right] \right\} \tag{S12}$$

$$\text{Residual}_{\mathrm{DMT}} = 100 - Yield_{\mathrm{DMT}} \tag{S13}$$

The calculation of DEA yields and residuals from polymerization reactions were similar and based on the relation with of overall adipate unit represented by integration value at 2.32 ppm ($I_{2.32}$), which represents 4 hydrogens, minus the residual of DEA marker at 1.26 ppm ($I_{1.26}$), representing 6 hydrogens. The following equations were employed to estimate the final yield and residual of DEA, respectively:

$$Yield_{\mathrm{DEA}} = 100 \cdot \left\{ I_{2.32} - \left[\left(4 \text{ hydrogens} \right) \cdot \left(\frac{I_{1.26}}{6 \text{ hydrogens}} \right) \right] \right\} \tag{S14}$$

$$\text{Residual}_{\mathrm{DEA}} = 100 - Yield_{\mathrm{DEA}} \tag{S15}$$

Life Cycle Assessment (LCA)

Table S9. Input and output data from LCA modeling for the production of 1 kg of PBAT under Cases A, B, and C, as modeled in OpenLCA.

	**Case A**	**Cases B and C**	**Unit**
**Input Flow (OpenLCA)**	**Value**		
Adipic acid	0.3690	0.2389	kg
Butane-1,4-diol	0.4580	0.0000	kg
Compressed air, 1200 kPa gauge	0.0644	0.0644	m^3
Electricity, medium voltage	0.4180	0.4180	kWh
Heat, district or industrial, other than natural gas	0.0403	0.0403	MJ
Nitrogen, liquid	0.0653	0.0653	kg
Purified terephthalic acid	0.3680	0.2716	kg
Steam, in chemical industry	0.3720	0.3720	kg
Tap water	3.8200	3.8200	kg
Transport, freight, lorry, unspecified	597.5	597.5	kg·km
Gas, natural, in ground	0.1360	0.1360	Nm^3
PBAT glycolysis	0.0000	0.4895	kg
**Output Flow (OpenLCA)**	**Value**		**Unit**
PBAT granulate	1.0000	1.0000	kg
Municipal solid waste	8.49E-04	8.49E-04	kg
1,4-Butanediol	7.14E-06	7.14E-06	kg
Ammonia	3.16E-08	3.16E-08	kg
Ammonia, as N	2.41E-06	2.41E-06	kg
BOD5, Biological Oxygen Demand	9.91E-06	9.91E-06	kg
Carbon monoxide	3.14E-07	3.14E-07	kg
COD, Chemical Oxygen Demand	3.98E-05	3.98E-05	kg
Furan, tetrahydro-	7.23E-05	7.23E-05	kg
Hazardous waste (deposited)	0.0065	0.0065	kg
Hydrogen sulfide	2.10E-08	2.10E-08	kg
Nitrogen oxides	1.80E-04	1.80E-04	kg
Nitrogen, total	1.04E-05	1.04E-05	kg
NMVOC, non-methane volatile organic compounds, unspecified origin	1.95E-04	1.95E-04	kg
Particulates	3.33E-05	3.33E-05	kg
Phosphorus, total	4.23E-07	4.23E-07	kg

Sulfur dioxide	2.52E-05	2.52E-05	kg
Suspended solids, unspecified	1.11E-05	1.11E-05	kg
Waste water	9.31E-04	9.31E-04	m^3

Table S10. LCA modeling input data for the production of 1 kg of PBAT glycolysis product under Cases B and C, as modeled in OpenLCA.

**Input Flow (OpenLCA)**	**Case B**	**Case C**	**Unit**
	**Value**		
Butane-1,4-diol	0.6319	0.6319	kg
PBAT granulate	0.3681	0.0000	kg
Tap water	1.0000	1.0000	kg
Electricity, medium voltage	4.3200	4.3200	kWh
Gas, natural, in ground	2.6500	2.6500	m^3
Transport, freight, lorry, unspecified	500	500	kg*km

Figure S3. Process flowcharts and system boundaries for LCA scenarios A, B, and C. A cradle-to-gate approach was adopted; therefore, the system boundaries are limited to the product leaving the factory gate. This includes raw material extraction, transport to the processing plant, and manufacturing, while explicitly excluding the product use and end-of-life disposal stages.

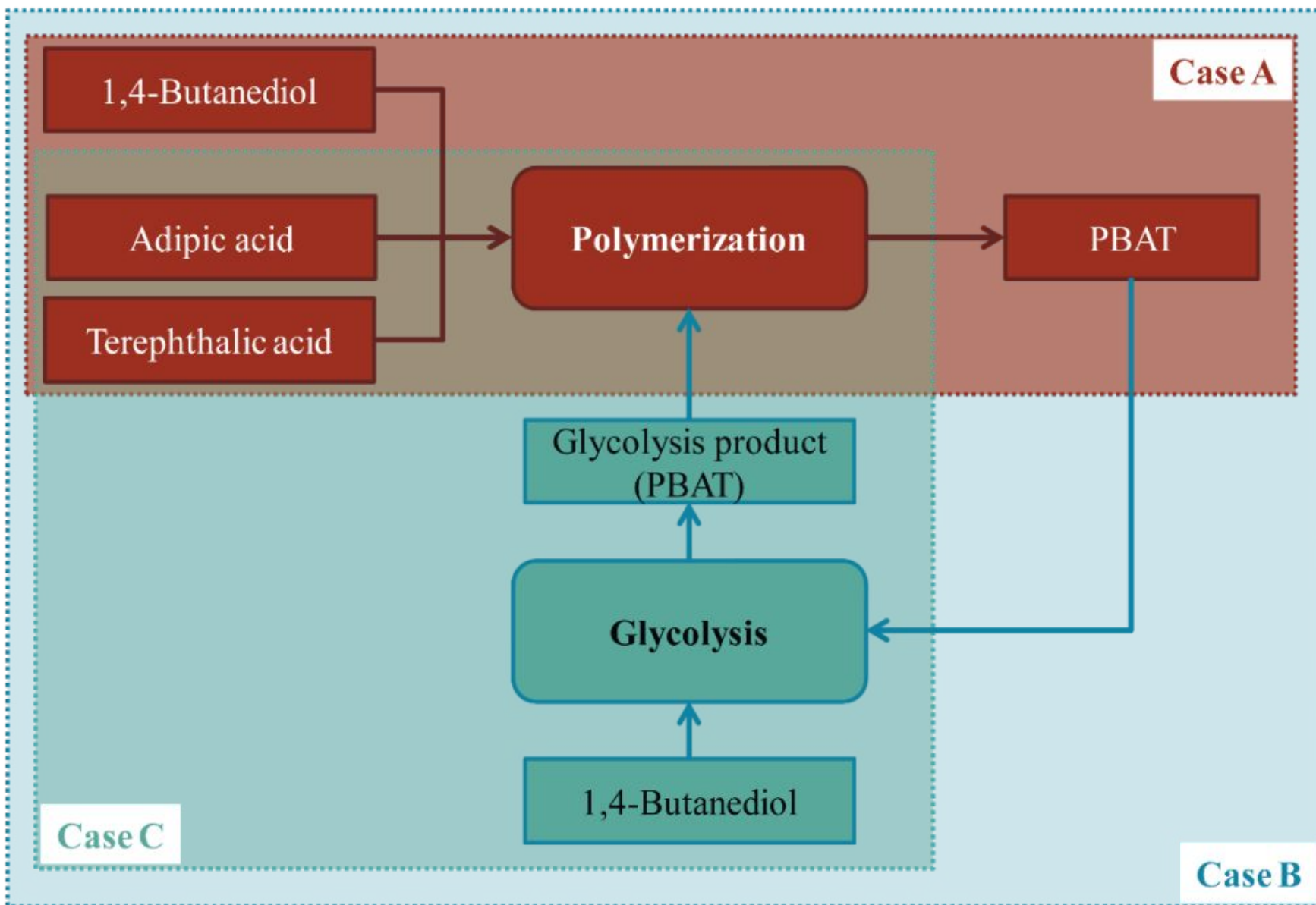

Results and Discussion

Glycolysis Reactions

Figure S4. Commercial PBAT characterization includes a) DSC; b) ^{1}HNMR; and c) WR GPC.

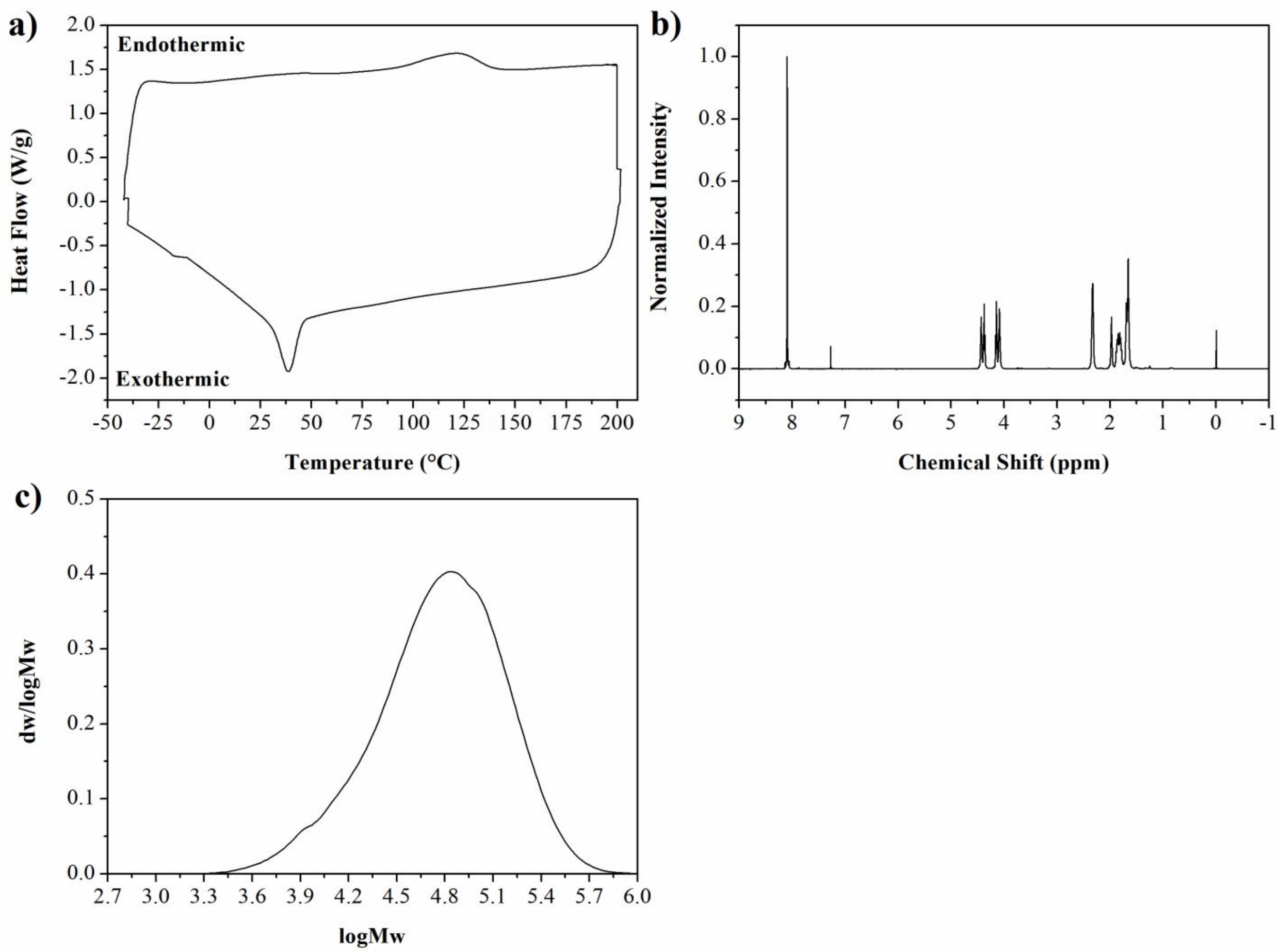

Figure S5. Normalized molar mass distribution obtained from low-molar-mass GPC, comparing reactions conducted with 0.70 mol% of $Sn(Bu)_2O$ (DP01 and DP04) and 5.00 mol% of $NaHCO_3$ (DP07 and DP08) using EG and 1,4-BD as glycols, respectively.

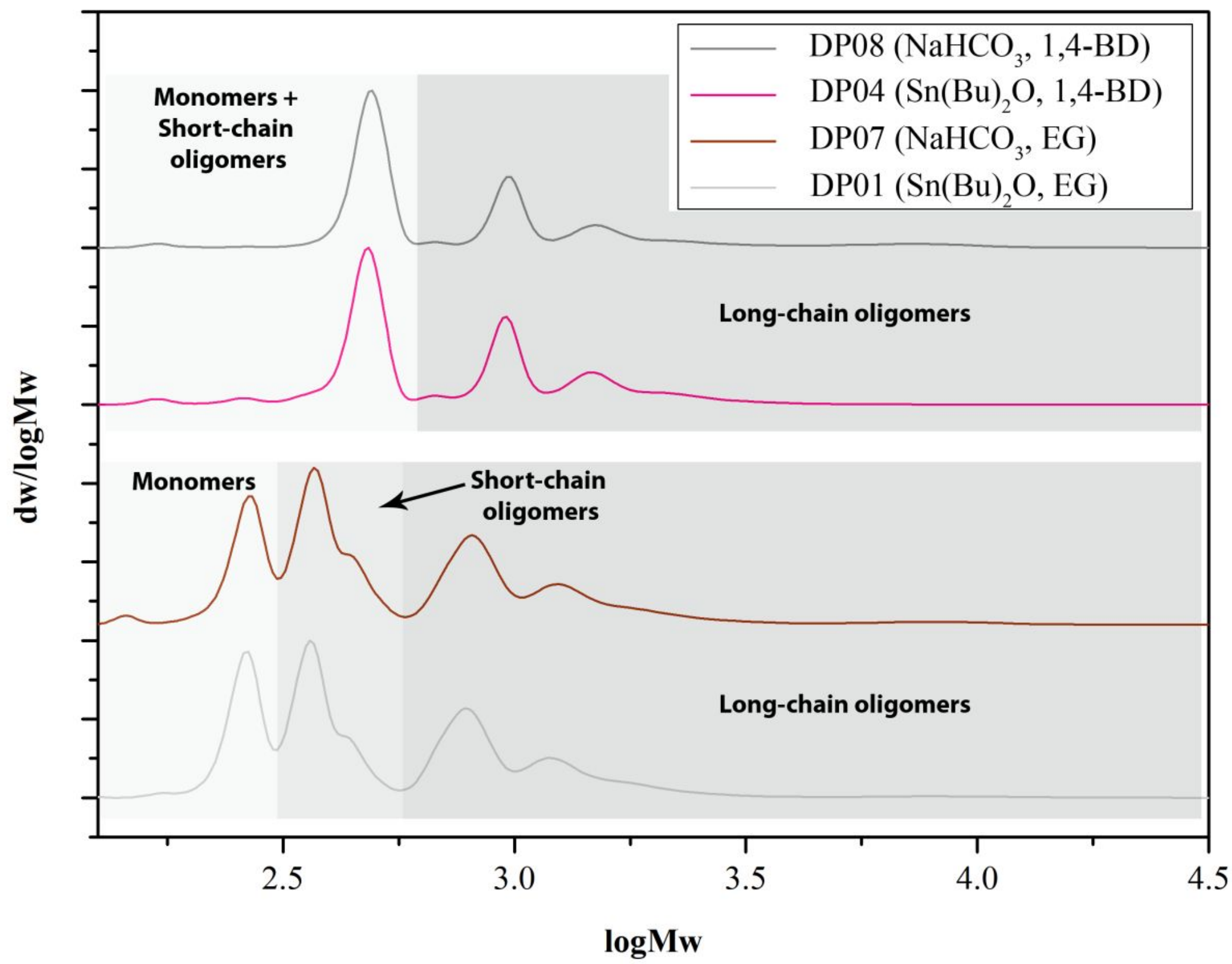

Figure S6. ¹HNMR spectra of glycolysis products (DP02, DP01, and DP03) obtained under identical catalyst concentration (0.70 mol% of $Sn(Bu)_2O$), reaction time (5 h), and glycol (EG) at temperatures of 150, 170, and 190 ˚C, respectively.

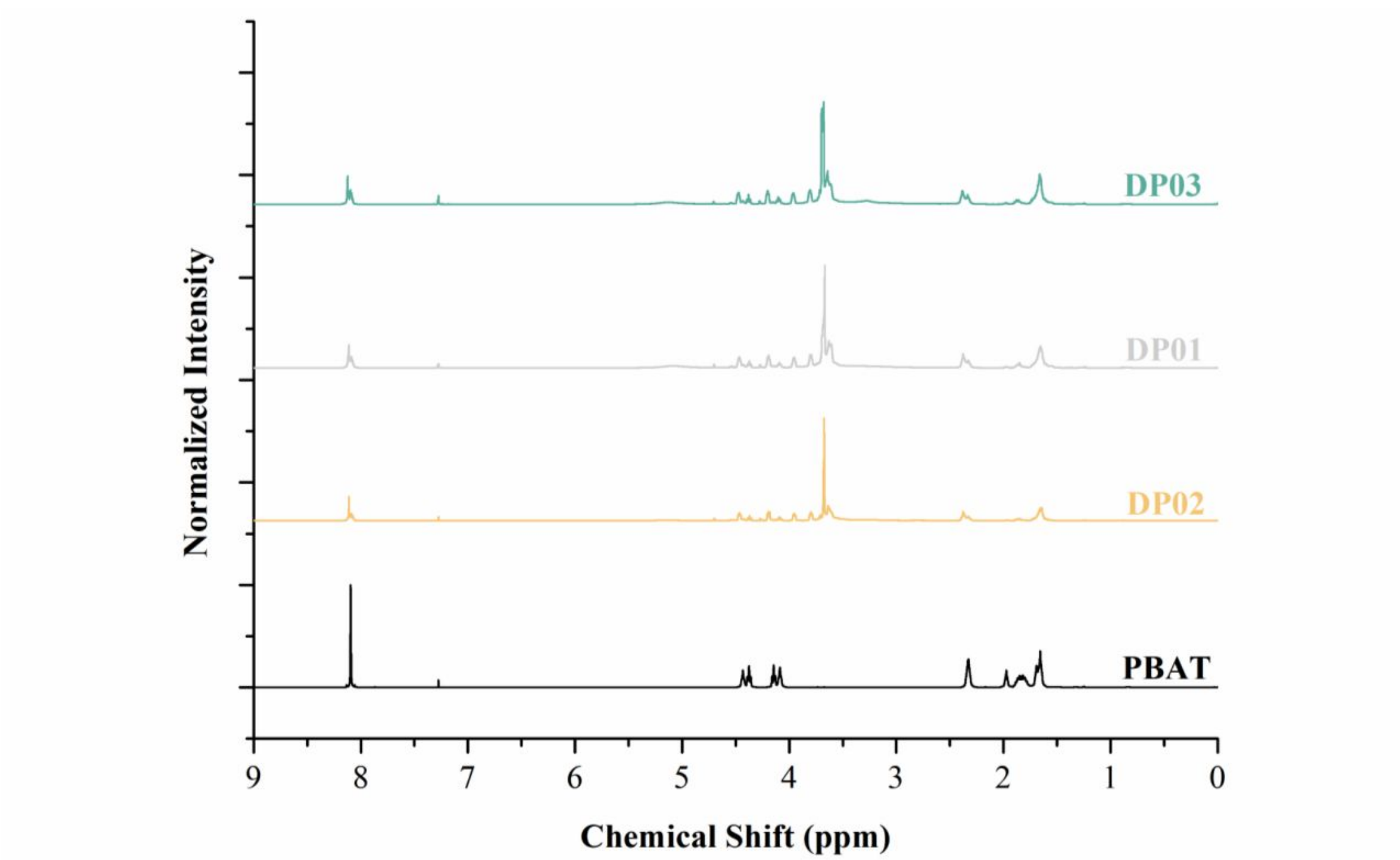

Figure S7. ^{1}HNMR spectra of glycolysis products (DP04 and DP08) obtained under identical glycol (1,4-BD), reaction time (5 h), and temperature (170 ˚C) with different catalyst systems consisting of 0.70 mol% of $Sn(Bu)_2O$ and 5.00 mol% of $NaHCO_3$, respectively.

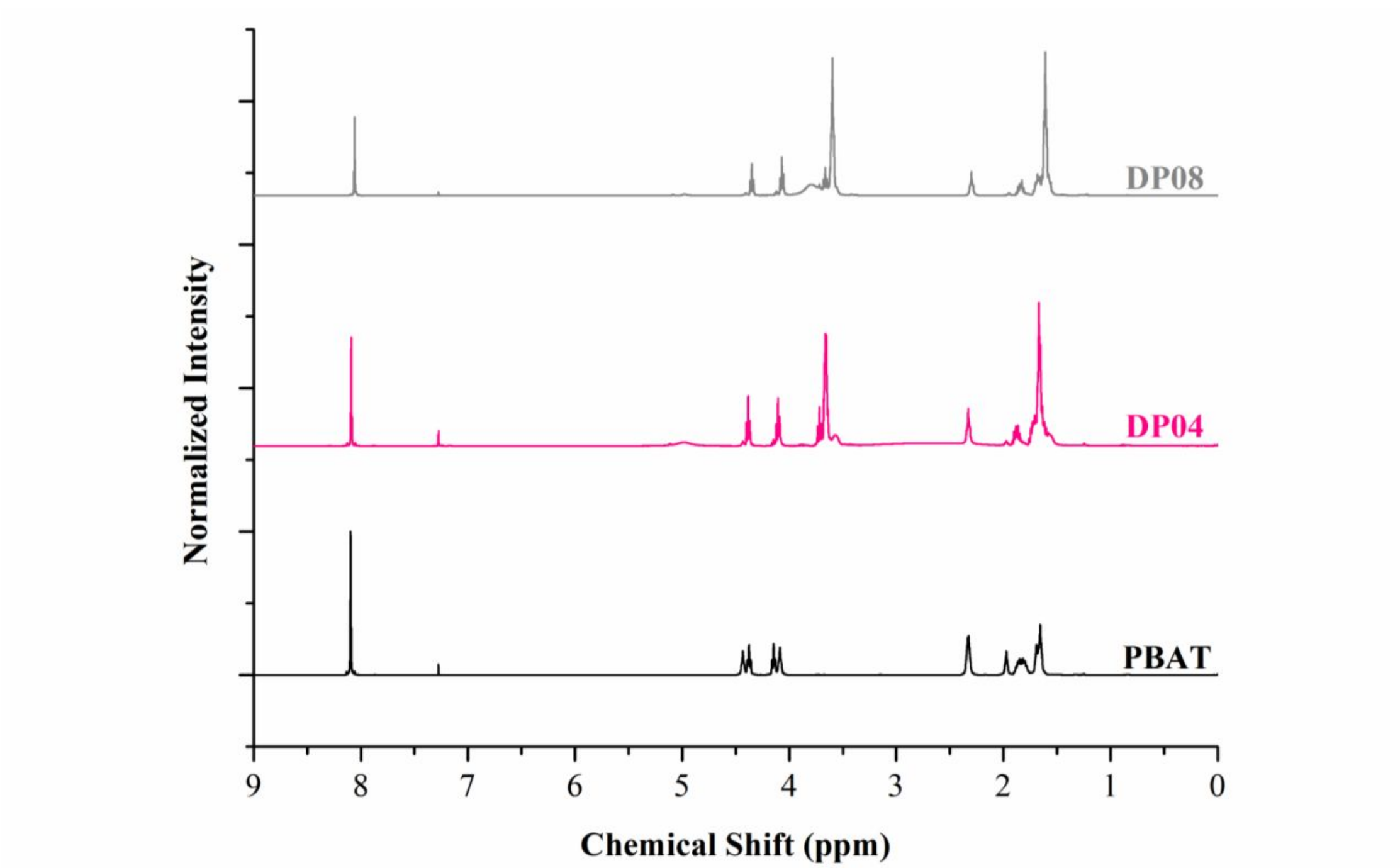

Polymerization Reactions

Evaluation of the polymerization of virgin raw materials was essential to establish a baseline for the experimental methodology, which would subsequently be adopted for the repolymerization of glycolysis products. The polycondensation results are summarized in Table 2. Initially, reactions were proposed using terephthalic and adipic acids as monomers, but the dispersion of terephthalic acid was hindered during P01, likely due to its low solubility in 1,4-BD and adipic acid. Consequently, subsequent polymerizations were conducted using the esters

DMT and DEA, along with 1,4-BD, as monomers. A $Ti(OBu)_4$ concentration of 0.2 mol% was maintained for all experiments.

As expected, the stoichiometric imbalance of DMT in reactions P02 and P03 resulted in lower molar masses compared to reactions P04 and P05, respectively. Furthermore, increasing the temperature from 190 to 210 ˚C during the vacuum stage did not promote chain growth; instead, it likely contributed to thermal-oxidative degradation, evidenced by the yellowing of the material. In contrast, reducing the reaction time under N2 atmosphere in favor of an extended vacuum period led to increased Mn and a narrower Đ, comparing reactions P05 and P07. This improvement is likely due to the enhanced removal of volatile byproducts (ethanol and methanol), driving the equilibrium toward higher molar masses.

Figure S8. ^{1}HNMR spectra of the repolymerization reaction products.

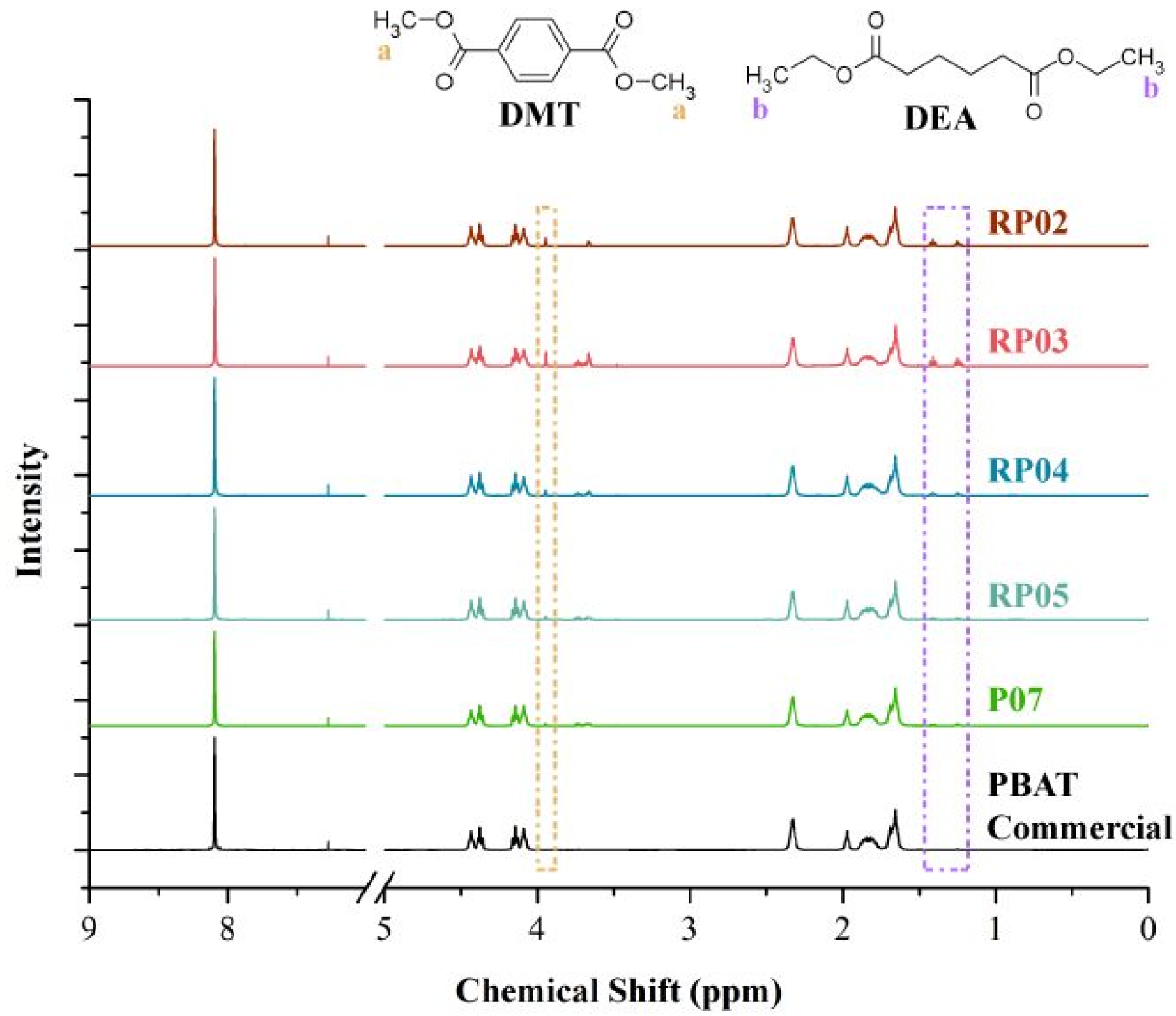
DMT
DEA
a
b
RP02
RP03
RP04
RP05
P07
PBAT Commercial
Intensity
Chemical Shift (ppm)
9
8
5
4
3
2
1
0
